# Exploring the Roles of Single Atom in Hydrogen Peroxide Photosynthesis

**DOI:** 10.1007/s40820-023-01231-1

**Published:** 2023-11-20

**Authors:** Kelin He, Zimo Huang, Chao Chen, Chuntian Qiu, Yu Lin Zhong, Qitao Zhang

**Affiliations:** 1 International Collaborative Laboratory of 2D Materials for Optoelectronics Science and Technology of Ministry of Education, Institute of Microscale Optoelectronics, Shenzhen University, Shenzhen, 518000 China; 2 Queensland Micro- and Nanotechnology Centre, School of Environment and Science, Griffith University, Nathan, QLD 4222 Australia; 3 Institute for Sustainable Transformation, School of Chemical Engineering and Light Industry, Guangdong University of Technology, Guangzhou, 51006 China; 4 ZJU-Hangzhou Global Scientific and Technological Innovation Center, Zhejiang University, Hangzhou, 311215 China

**Keywords:** Single atom catalysts, H_2_O_2_ photosynthesis, Catalyst design and optimization

## Abstract

This comprehensive review provides a deep exploration of the unique roles of single atom catalysts (SACs) in photocatalytic hydrogen peroxide (H_2_O_2_) production. SACs offer multiple benefits over traditional catalysts such as improved efficiency, selectivity, and flexibility due to their distinct electronic structure and unique properties. The review discusses the critical elements in the design of SACs, including the choice of metal atom, host material, and coordination environment, and how these elements impact the catalytic activity. The role of single atoms in photocatalytic H_2_O_2_ production is also analysed, focusing on enhancing light absorption and charge generation, improving the migration and separation of charge carriers, and lowering the energy barrier of adsorption and activation of reactants. Despite these advantages, several challenges, including H_2_O_2_ decomposition, stability of SACs, unclear mechanism, and low selectivity, need to be overcome. Looking towards the future, the review suggests promising research directions such as direct utilization of H_2_O_2_, high-throughput synthesis and screening, the creation of dual active sites, and employing density functional theory for investigating the mechanisms of SACs in H_2_O_2_ photosynthesis. This review provides valuable insights into the potential of single atom catalysts for advancing the field of photocatalytic H_2_O_2_ production.

## Highlights

- The review explores single atom catalysts (SACs) for photocatalytic H_2_O_2_ production, highlighting their unique structure, properties, and advantages over traditional catalysts. It emphasizes the importance of metal atom types, host material selection, and coordination environment in SACs design.
- The article explains how SACs enhance photocatalytic H_2_O_2_ production by improving light absorption, charge generation, migration, and lowering energy barriers for reactant adsorption and activation.
- The review acknowledges challenges and future research directions in SACs for H_2_O_2_ photosynthesis.

## Graphical abstract

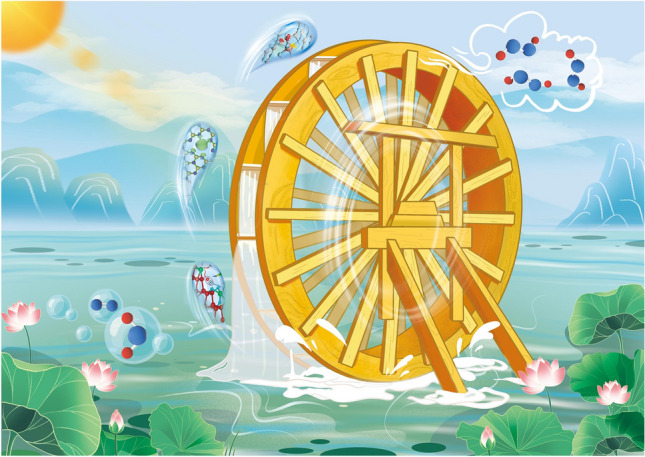

## Introduction

Hydrogen peroxide (H_2_O_2_) is an essential commodity chemical with diverse applications across industries including medicine, food processing, wastewater treatment, and paper and pulp manufacturing [1–3]. Traditional synthesis methods for H_2_O_2_ involve intricate multi-step pathways, coupled with substantial energy consumption and reliance on hazardous materials, culminating in significant environmental and economic impediments. Consequently, there has been a marked shift towards photocatalytic production of H_2_O_2_, attracted by its environmentally benign and sustainable characteristics. In the process of photocatalysis, a photocatalyst absorbs photons, generating electron–hole pairs. These pairs then engage in redox reactions with surrounding substrates, leading to the formation of value-added products such as H_2_O_2_ [4–7]. However, conventional photocatalysts have been constrained by limitations including restricted light absorption, inefficient charge separation, and low selectivity, hindering the overall efficacy of H_2_O_2_ production [8–11]. This underscores the necessity for pioneering novel photocatalytic materials endowed with enhanced properties.

Single atom catalysts (SACs), which have come to prominence in recent years, present an appealing class of photocatalysts [12–14]. Comprising individual atoms (predominantly non-noble metals) dispersed on compatible support materials, each atom functions as an active site. The atomic-level dispersion inherent to SACs ensures optimal atom utilization, fostering remarkable catalytic activity and selectivity [15, 16]. Beyond the conventional scope of photocatalytic H_2_O_2_ production, Single atom catalysts (SACs) have emerged as a versatile tool in diverse fields such as energy conversion, organic synthesis, and environmental remediation. In the realm of energy conversion, SACs have been instrumental in the electrochemical reduction of CO_2_ to essential hydrocarbons, hydrogen evolution, and fuel cell applications, showing enhanced catalytic activity and a reduction in overpotentials. This innovation represents a significant advancement in the field. In organic synthesis, the implementation of SACs has led to notable improvements in reaction kinetics, including accelerated reaction rates and superior selectivity. This effect is particularly pronounced in hydrogenation and oxidation reactions, where the unique characteristics of SACs provide an unprecedented level of efficiency and control. Additionally, in the automotive and industrial contexts, SACs have been employed to effectively convert pollutants such as NO_x_, CO, and hydrocarbons into innocuous end-products [11]. This application of SACs constitutes a pivotal step towards a more sustainable and cleaner environment, reinforcing the importance of continued exploration and exploitation of these catalysts in various scientific and technological domains.

While there is growing interest in the field of single atom photocatalysis, a comprehensive understanding of the mechanisms guiding the performance of these catalysts, especially in the context of photocatalytic H_2_O_2_ production. This review undertakes a comprehensive examination of the fundamentals of SACs, focusing on their distinctive properties, comparative advantages over traditional catalysts, and the crucial factors that influence their design. It further delves into the specific reaction pathways, characterization methodologies, synthesis approaches, and the complex roles single atoms play in photocatalytic H_2_O_2_ production. In addressing existing challenges and outlining potential strategies for prospective research directions, the review presents an integrative perspective that accentuates the importance of SACs in the evolving landscape of catalytic science, while also establishing forth a conceptual framework for future progression. The subsequent sections will explore the nuanced details of SACs, laying the groundwork for a robust understanding that could steer upcoming innovations in the realms of sustainable chemistry and industrial applications.

## Fundamentals of Single Atom Catalysis

Single atom catalysis is a cutting-edge concept in catalytic processes that revolves around the use of single atoms, typically metals, as the active catalytic sites [17]. This concept deviates from the traditional catalytic models involving metal clusters or antiparticle, focusing instead on individual atoms that act as discrete, active sites for catalysis [18].

### Definition and Properties of Single Atom Catalysts

SACs are catalysts where individual atoms, typically metals, act as the catalytic active sites [19]. Unlike in traditional catalysts, where atoms are aggregated, these individual atoms are robustly anchored onto a substrate. This anchoring prevents aggregation or detachment and ensures the catalyst's stability [20, 21]. Each single atom serves as a unique active site, interacting with reactants at the atomic scale.

**Fig. 1:**
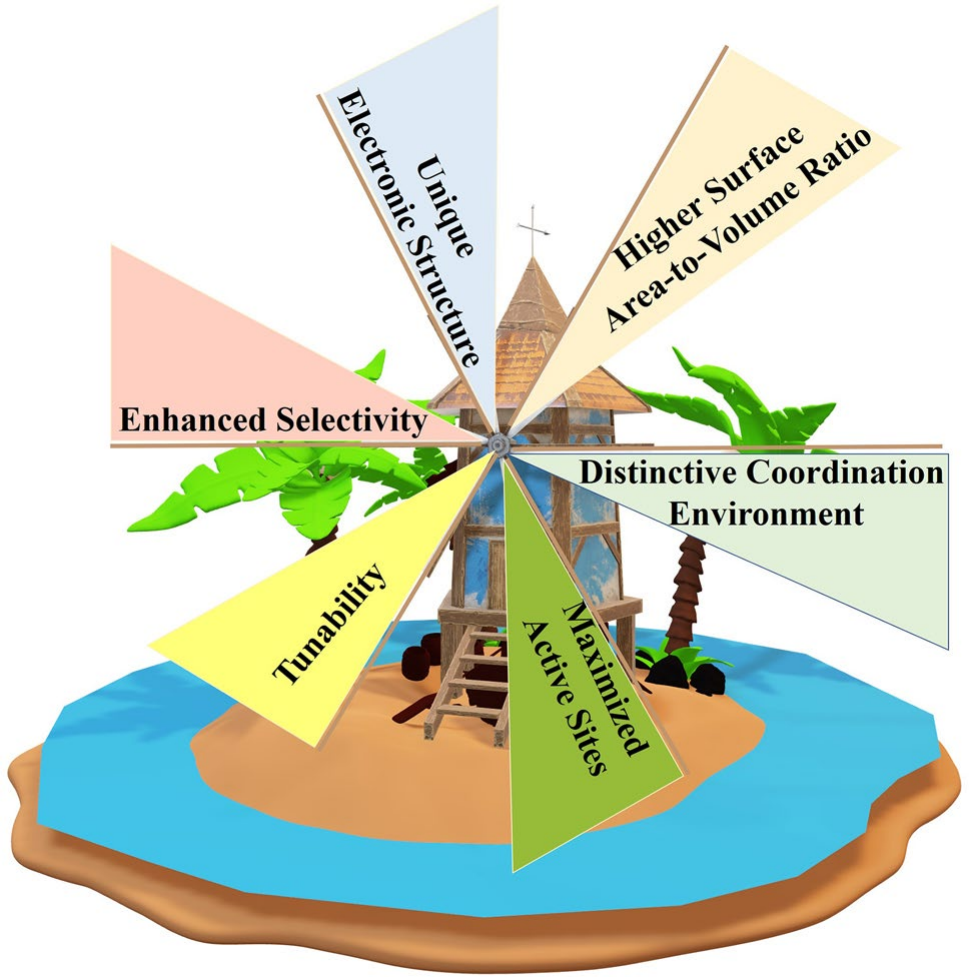
Advantages of single atom catalysts over these traditional catalysts

The isolated nature of these atoms means that their electronic structure—the distribution and energy of their electrons—differs greatly from that of bulk metals or clusters [22]. This distinct configuration endows SACs with novel catalytic properties, potentially enhancing their activity, selectivity, and stability, particularly in the context of H_2_O_2_ photosynthesis (Fig. 1).

### Advantages of SACs over Traditional Catalysts

#### Reactivity

Reactivity, a defining characteristic of modern catalytic systems, plays a pivotal role in determining the overall efficiency and effectiveness of chemical processes [23]. SACs have shown remarkable potential in this domain, and this section explores the underlying features that contribute to their enhanced reactivity compared to traditional bulk or nanoparticle catalysts.

In SACs, the distinctiveness arises from isolating individual atoms; here, every atom has the potential to serve as an active site. This unique configuration provides higher accessibility to the reactive centers, facilitating more frequent and effective interactions with reactants [14]. The particular coordination environment surrounding these isolated atoms gives rise to unique electronic structures, further enhancing the catalytic activities. This combination of structural isolation and distinct electronic properties has been pivotal in elevating the reactivity of SACs significantly, leading to promising applications in various catalytic processes. On the other hand, traditional catalysts composed of bulk materials or nanoparticles often face inherent limitations in reactivity. The clustering or aggregation of atoms in these systems can lead to less accessible active sites, impeding the interaction with reactants [24]. This reduced accessibility to active sites inevitably diminishes the overall reactivity of the catalyst, thereby constraining the efficiency of the catalytic process.

In conclusion, the enhanced reactivity exhibited by SACs underscores their potential as a groundbreaking advancement in the field of catalysis. By utilizing the isolation of individual atoms and leveraging unique electronic structures, SACs offer a clear advantage in terms of reactivity over traditional bulk or nanoparticle catalysts. This distinction not only highlights the innovative nature of SACs but also encourages further exploration and development to harness their full potential across a wide array of industrial applications. Future research in this area may focus on understanding the specific factors influencing reactivity in SACs and optimizing their design for various applications.

#### Selectivity

The domain of catalysis is continually seeking innovative approaches to achieve greater selectivity in reactions. In this realm, SACs have emerged as a promising tool, providing unique characteristics that lead to superior selectivity for desired reaction pathways [25]. This section elucidates the enhanced selectivity of SACs, focusing on the specific application of H_2_O_2_ photosynthesis, to provide an insight into the nuanced advantages over traditional catalysts.

Traditional catalysts, with their bulk structures, may encounter limitations in distinguishing between different reaction pathways effectively. Limited selectivity can lead to unwanted by-products, requiring intricate, expensive separation processes and potentially reducing reaction efficiency [26]. Conversely, SACs, owing to their unique electronic structure, have demonstrated the ability to offer enhanced selectivity in catalytic reactions. Each atom in an SAC serves as an independent active site, providing a distinctive electronic environment that can selectively promote certain reaction pathways over others. In the context of H_2_O_2_ photosynthesis, this heightened selectivity becomes particularly significant [23]. The application of SACs in this process can result in a higher yield of H_2_O_2_, with fewer impurities and side reactions. This not only enhances the efficiency of the process but also contributes to the purity of the final product.

In conclusion, the enhanced selectivity of SACs represents a groundbreaking advancement in catalysis, offering substantial benefits over traditional catalyst systems. The unique electronic structure of SACs allows for more precise control over reaction pathways, thereby improving yields and minimizing unwanted by-products. This feature holds considerable promise for various industrial applications and warrants further research to explore the full potential of SACs in modern catalytic processes. Future studies may also consider exploring other attributes of SACs, including stability, scalability, and integration into existing industrial frameworks.

#### Tunability

Tunability, in the context of catalysis, refers to the capacity to adjust and control specific properties to achieve desired performance characteristics. This concept is central to catalyst design and optimization, especially given the varying requirements of chemical reaction [27]. This section elucidates the stark contrast between the tunability offered by SACs and traditional catalysts.

Single atom catalysts present a promising frontier in the field of catalysis due to their enhanced tunability. The isolation of individual atoms in SACs permits an unparalleled influence over their electronic properties. This influence is facilitated by distinct coordination environments that allow for precise modulation of electronic structures. As a result, SACs can be finely tailored, bolstering reactivity and selectivity to meet specific process requirements [28]. This capacity for customization is not just theoretically appealing but translates into practical benefits, with potential applications in various industrial sectors. In contrast, the tuning of electronic properties in traditional catalysts remains a more complex and challenging endeavour. The structural complexity of traditional catalysts, often involving aggregated or clustered atoms, creates multifaceted electronic environments that are more resistant to precise control. Attempting to alter specific electronic properties in such a heterogeneous system may lead to unintended interactions and conflicting effects, hindering the ability to achieve clear-cut customization [29]. This inherent complexity thereby limits the flexibility and precision with which traditional catalysts can be adapted to specialized reactions.

To conclude, the ability to tune the electronic properties of catalysts opens up vast possibilities for innovation and optimization in the field of catalysis. While SACs demonstrate a distinct advantage in this regard, offering precise control and adaptability, traditional catalysts present more challenges due to their inherent structural complexity. The ongoing exploration of the tunability of SACs promises exciting prospects for the development of novel, tailor-made catalytic systems. Further research into the intricate coordination environments of SACs, along with advancements in synthesis and characterization techniques, is anticipated to drive further progress and innovation in this dynamic area of study.

#### Surface Area-to-Volume Ratio

In the arena of catalytic processes, the optimization of surface area-to-volume ratio stands as a critical factor in enhancing efficiency [30]. This paper explores the remarkable characteristics of SACs concerning this aspect, contrasting them with traditional catalyst systems, which predominantly consist of larger particles or clusters of atoms.

Conventional catalysts, due to their bulkier structures, commonly face challenges in optimizing the surface area accessible to reactants. The particles or clusters of atoms constituting the catalytic site may result in lower surface area-to-volume ratios [31]. This structure inherently restricts the availability of catalytic material for interaction with reactants, thus influencing the efficiency of the catalytic reaction negatively. In stark contrast, SACs introduce a novel architecture that leverages a higher surface area-to-volume ratio. With SACs, each individual atom is exposed, acting as an independent active site. This unique configuration ensures that virtually the entire surface of the catalyst is available for interaction with reactants [31]. Consequently, the surface area-to-volume ratio in SACs is substantially increased, providing a platform for enhanced catalytic efficiency. The implications of this higher surface area-to-volume ratio in SACs extend beyond mere geometric considerations. The increased availability of catalytic material amplifies the opportunities for interaction with reactants, potentially leading to higher activity, selectivity, and reaction rates [32]. This characteristic of SACs could pave the way for diverse industrial applications, championing efficiency and eco-friendliness.

In conclusion, the inherent structure of SACs, characterized by a higher surface area-to-volume ratio, represents a significant advancement in catalysis. This attribute, setting SACs apart from traditional catalyst systems, introduces a new dimension in the field of catalysis, emphasizing efficiency, and offers promising prospects for further research and industrial applications. Future investigations may focus on the synthesis, stability, and integration of SACs in various processes, contributing to a more comprehensive understanding of their potential in modern catalysis.

#### Stability

In catalysis, stability refers to the ability of a catalyst to maintain its structural integrity and activity under the specific conditions of a reaction. Stability is paramount in determining the lifespan and reliability of a catalyst, thus having a direct impact on its commercial viability and environmental footprint [33]. This section delves into the contrasting stability profiles of SACs and traditional nanoparticle catalysts, highlighting the key determinants of this vital property.

The innovative design of SACs, where single atoms are anchored to a support material, offers a unique solution to the challenges of stability. This anchoring counteracts common degradation mechanisms, such as sintering or aggregation, that can occur under various reaction conditions. By mitigating these destabilizing processes, SACs preserve their distinctive surface properties and active sites, guaranteeing consistent performance over extended periods [34]. The development of robust anchoring methods and the careful selection of support materials have been central to leveraging the stability advantages of SACs, contributing to their growing prominence in both research and industrial applications. Conversely, nanoparticle-based traditional catalysts grapple with innate stability issues. Over time, these nanoparticles may undergo agglomeration, leading to a loss of their specific surface properties. This agglomeration reduces the available active sites and alters the electronic structure, thereby diminishing the catalyst's overall effectiveness and efficiency [35]. The propensity for agglomeration and subsequent degradation is influenced by factors such as particle size, support interaction, and reaction environment, requiring careful consideration in the design and operation of traditional catalysts.

In summary, stability in catalysts is a multifaceted property that hinges on a complex interplay of structural, chemical, and operational factors. The comparative analysis of SACs and traditional nanoparticle catalysts underscores the inherent advantage of SACs in maintaining stability. By utilizing anchoring techniques and optimizing support materials, SACs exhibit resilience against common degradation pathways, affirming their potential as a next-generation catalytic solution. Conversely, the stability challenges faced by traditional catalysts necessitate ongoing research and innovation to mitigate degradation and extend their functional lifespan. Ongoing investigations into stability mechanisms, paired with strides in materials science, are set to influence catalysis' future direction, marrying technological advancements with sustainable goals.

#### Cost

Economic considerations, specifically cost reduction, are paramount in the field of catalysis, influencing not only research and development but also industrial application and sustainability [36]. This section delves into the cost implications of SACs versus traditional catalysts, highlighting the nuances that contribute to their respective economic profiles.

SACs herald a paradigm shift in material efficiency by allowing every atom to serve as an active site. Such maximized material utilization paves the way for significant cost savings, especially pertinent when dealing with precious metals [37]. By ensuring that every single atom can participate in the reaction, SACs optimize material usage, translating this efficiency into a tangible economic advantage. However, it is essential to recognize that the synthesis and preparation of SACs might entail more complexity and initial expense. Yet, this upfront investment can be outweighed by the long-term benefits of material efficiency, leading to overall cost savings. The financial appeal of SACs is, therefore, intricately tied to their innovative design and judicious use of materials, aligning cost effectiveness with top-tier catalytic performance [25]. In contrast, traditional catalysts, characterized by clusters or bulk structures, present challenges in material utilization. A substantial portion of the material may remain unexposed to reactants and consequently inactive in catalysis. This inefficiency can inflate costs, especially when expensive materials are involved. Furthermore, the requirement for larger quantities of material might exacerbate costs without corresponding increases in catalytic activity [25]. This disproportionality between material usage and performance raises critical questions about the cost effectiveness of traditional catalysts, emphasizing the need for thoughtful design and optimization.

In conclusion, the contrast between SACs and traditional catalysts in terms of cost reveals a complex interplay of factors such as material efficiency, synthesis complexity, and performance optimization. SACs, with their unique ability to leverage every atom, offer a promising avenue for cost reduction, especially in the context of precious metals. Traditional catalysts, on the other hand, must grapple with inherent inefficiencies that can impact their overall cost profile. The perspectives drawn from this juxtaposition accentuate the layered considerations surrounding catalysis costs. Such insights serve as a beacon for researchers, engineers, and decision-makers charting the shifting terrains of catalytic innovations.

### Critical Elements in Single Atom Catalyst Design

Designing an effective single atom catalyst requires a meticulous consideration of various components that determine the catalyst's performance [38]. Here, we delve into three key aspects that are essential in the design: types of metal atom, host materials, and coordination environments.

#### Types of Metal Atom

**Fig. 2:**
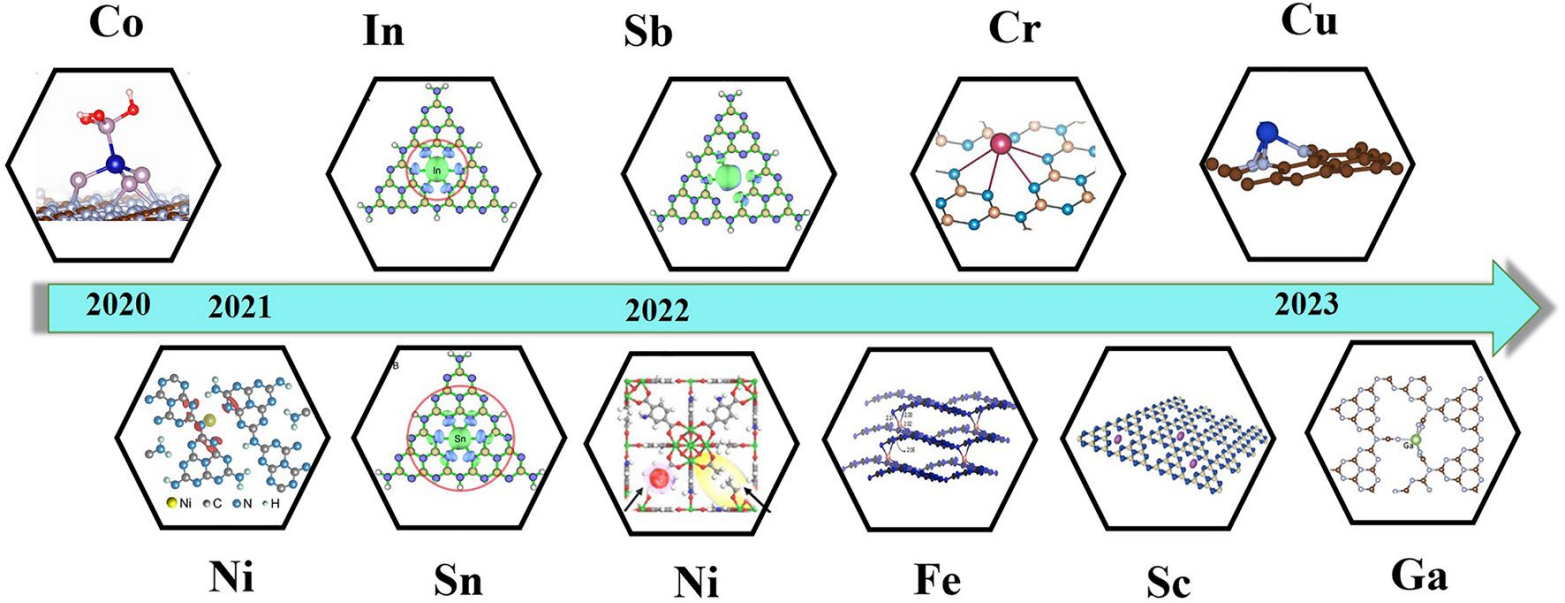
Brief timeline of non-noble metals single-atom catalysts for H_2_O_2_ photosynthesis, [41] Copyright 2023, Springer Nature; [46] Copyright 2022, Wiley-VCH; [47] Copyright 2022, Elsevier; [107] Copyright 2021, Springer Nature; [103] Copyright 2021, Royal Society of Chemistry; [26] Copyright 2020, PNAS; [42] Copyright 2023, Elsevier; [44] Copyright 2022, Springer Nature; [117] Copyright 2021, Elsevier; [27] Copyright 2022, American Chemical Society

Transition metal single atom catalysts refer to single atom catalysts where the metal atom is a transition metal, such as Fe, Ni, Co, Cu, Sc, Ga, or Cr (Fig. 2) [39–47]. Non-noble metals typically have multiple oxidation states and are known for their ability to facilitate a wide range of chemical transformations. They are generally abundant and less expensive, making them attractive candidates for catalyst design. Noble metal single atom catalysts refer to single atom catalysts where the metal atom is a noble metal, such as Au, Ag, Pt, or Pd [48–51]. Noble metals are known for their excellent catalytic properties, including high activity and selectivity. However, they are less abundant and more expensive than non-noble metals.

Noble metals generally exhibit superior catalytic activity compared to their non-noble counterparts. This is largely due to their ability to efficiently adsorb reactants and lower the activation energy for reactions. In noble metals, the d-band centre lies closer to the Fermi level compared to non-noble metals, facilitating the transfer of electrons during catalysis [52, 53]. This promotes catalytic activity by reducing energy barriers and accelerating reaction rates. Noble metals have high electronegativity and ionization energy [54]. These characteristics enable noble metals to form stronger bonds with adsorbates, enhancing catalytic activity by improving reactant adsorption and activation [55].

Non-noble metals can exhibit a greater diversity in catalytic transformations due to the variability in their oxidation states, which allows for more flexibility in tuning the catalytic process [56–58]. Non-noble metals have multiple oxidation states due to the unique arrangement of their d-electrons. The ability to readily gain and lose electrons means they can be a part of various oxidation–reduction reactions. The stability of a given oxidation state depends on the specific reaction environment, including the presence of other reactive species. As an illustration, the research spearheaded by Xu's team delves into the application of iron single-atoms (FeSA) with oscillating oxidation states. These states are realized via diverse precursors and are ensconced within a matrix of nitrogen-enriched functionalized carbon quantum dots (CQDs) matrix [59]. They explore these alternative catalytic pathways experimentally and theoretically, aiming to gain a comprehensive understanding of the redox chemistry that drives the FeSA@CQDs system. It is found that these alternative catalytic pathways are dependent on the oxidation states of the FeSAs.

#### Support Material

The role of the support materials in SACs is fundamental. They serve as the host for the single metal atoms and significantly influence the electronic structure and catalytic performance of the metal atoms [60]. The nature of the support can tailor the electronic properties of the metal atoms, modulate their interaction with the reactants, and stabilize the single atom structure, which are all essential factors for the photocatalytic H_2_O_2_ production [17].

The support material provides a stable environment to anchor the single atom, preserving its single atom status and preventing it from agglomerating with other atoms, which could improve its catalytic activity [61]. The stability and anti-agglomeration of single atom catalysts are influenced by the properties of their host materials, such as strong metal-support interaction, surface chemistry, structural confinement, electronic structure, and matching lattice parameters [62]. These attributes help maintain the independent status of single atoms, preventing them from forming larger particles. The host materials provide strong anchoring effects, chemical functionality, geometric structure, electronic environments, and lattice parameters compatible with atomic radius to stabilize single atoms [63]. Researchers can utilize these features to design effective host materials for single atom catalysts, enhancing their catalytic potential. From Table 1, the study of support materials mainly focuses on graphitic carbon nitride in hydrogen peroxide photosynthesis. Graphitic carbon nitride (g-C_3_N_4_), a carbon-based material, has several advantageous features that make it a popular choice in the synthesis of single atom catalysts for photocatalytic H_2_O_2_ production [64]. Graphitic carbon nitride has a rich nitrogen chemistry which allows for the creation of active sites by functionalizing with different types of single atoms [65–67]. Its structure, characterized by tri-*s*-triazine units connected by amino groups, provides numerous sites for anchoring metal atoms, allowing for high dispersion of single atom catalysts on its surface [68, 69]. Furthermore, g-C_3_N_4_ is thermally stable up to 600 °C, which is beneficial during the catalyst synthesis and photocatalytic process. The high thermal stability ensures that the structure of the single atom catalyst and the support remains intact, preserving the catalytic activity and selectivity [70]. Moreover, the surface properties of g-C_3_N_4_ can be easily modified to facilitate the coordination of single atoms and tune the electronic properties of the catalysts. This can be achieved, for example, by introducing defects or doping with heteroatoms. Lastly, g-C_3_N_4_ is easily synthesized from low-cost and widely available precursors such as urea or melamine [11, 71]. The simplicity and cost-effectiveness of its synthesis process make it an attractive choice for photocatalyst support material.

**Table 1:** Summary of single atom catalysts in hydrogen peroxide photosynthesis

Metal/support materials	Synthesis method	Coordination	Efficiency (mmol h^−1^ g^−1^)	References
Co/C_3_N_4_@GO	Pyrolysis	S–Co–(N)_3_	16.58	[40]
Co/C_3_N_4_	Pyrolysis	Co–(N)_2_	0.003	[117]
Co/C_3_N_4_	Impregnation	Co–(P)_4_	10.3	[26]
Sb/C_3_N_4_	Pyrolysis	Sb–(N)_4_	0.091	[107]
Fe/C_3_N_4_	Pyrolysis	Fe–(N)_3_	0.02	[117]
Fe/C_3_N_4_	Pyrolysis	(N)_2_–Fe–(O)_2_	40.19	[39]
Ni/C_3_N_4_	Pyrolysis	Ni–(N)_2_	0.035	[117]
Ni/C_3_N_4_	Pyrolysis	Ni–N	0.024	[103]
Ni/C_3_N_4_	Impregnation	Ni–(N)_4_	27.11	[43]
Ni/red phosphorus	Photo-deposition	(P–O)_4_–Ni	2.288	[45]
Ni/UiO–66–NH_2_	Impregnation	Ni–N(O)	0.225	[27]
Mn/C_3_N_4_	Pyrolysis	(O)_4_–Mn–(N)_2_	7.964	[173]
In/C_3_N_4_	Pyrolysis	In–(N)_6_	0.2	[117]
Sn/C_3_N_4_	Pyrolysis	Sn–(N)_6_	0.12	[117]
Cu/N doped graphene-C_3_N_4_	Pyrolysis	Cu–(N)_3_	2.856	[42]
Sc/C_3_N_4_	Pyrolysis	Sc–(N)_3_	1.1	[44]
Cr/C_3_N_4_	Pyrolysis	Cr–(N)_4_	13.88	[46]
Pd/NH_2_–UiO–66	Impregnation	Pd–(O)_2_	1.74	[106]
Ga/C_3_N_4_	Pyrolysis	Ga–(N)_4_	0.332	[41]
Pt/CdS	Photoreduction	Pt–S	251	[73]
Ru/P doped C_3_N_4_	Photoreduction	Ru–(N)_2_	0.388	[48]

Various support materials present unique properties that render them promising candidates for SACs. Cadmium sulfide (CdS), for instance, is notable for its ability to provide distinct anchoring sites for single atoms, complemented by a controllable electronic environment [72]. Its inherent proficiency in visible-light absorption considerably enhances its potential for photocatalytic activity. Sharma et al. demonstrated the synthesis of isolated Platinum single-atoms (PtSA) anchored on Cadmium Sulfide nanoparticles (CdSIS), forming a well-dispersed configuration known as PtSA-CdSIS [73]. EXAFS analysis validates that Platinum remains atomically dispersed on the in-situ-generated CdSIS, manifesting a unique coordination geometry that is vital for catalytic performance. The Pt–S coordination, high BET-specific surface area, and mesoporous character of in-situ generated CdSIS contribute to superior light harvesting and catalytic active sites, thus promoting exceptional catalytic activity, selectivity, and efficient utilization of photoactive charge carriers. XPS spectra insights highlight a fortified electronic interaction between PtSA and CdSIS, enhancing electron mobility across the interface and minimizing energy barriers for H_2_O_2_ and hydrogen (H_2_) evolution. Beyond CdS, UiO–66–NH_2_ utilized as a support for single-atom nickel (Ni) in H_2_O_2_ photosynthesis unveils a synergistic design with distinct characteristics [27]. In the UiO–66–NH_2_ structure, amino groups (–NH_2_) robustly coordinate with nickel species, an interaction confirmed by FT-IR and N 1*s* XPS measurements that reveal atomically dispersed Ni single atoms within the MOF matrix. This interaction stabilizes Ni species, fostering efficient transfer and utilization of photoexcited carriers. Photoluminescence (PL) emission spectroscopy emphasizes the suppression of electron–hole pair recombination in the presence of Ni, thus improving photocatalytic activity. The UiO–66–NH_2_ structure, in conjunction with missing-linker defects and Ni single atoms, induces a synergistic effect that substantially amplifies H_2_O_2_ production. This leads to effective utilization of photogenerated charges and suppression of H_2_O_2_ decomposition. The selective two-electron water oxidation, catalyzed by Ni single atoms, boasts a high selectivity of 86% for H_2_O_2_ production. The inclusion of Ni in the Hf–UiO–66–NH_2_ catalyst enhances H_2_O_2_ production by 3.1-fold under visible-light irradiation, validating Ni as an optimal metal cocatalyst species. Notably, depositing Ni species into the Hf-0.5 MOF maintains its crystallinity and porosity, facilitating its role in catalytic reactions. The tailored band structures of the modified MOFs are favourable for photocatalytic H_2_O_2_ production from O_2_ and water, with Ni single atoms facilitating photogenerated hole migration. The role of Sc^3+^ ions in boosting H_2_O_2_ production is also highlighted, showcasing a complementary effect that augments the efficiency of the Ni/Hf-0.5 system. The comprehensive insights establish UiO–66–NH_2_'s suitability as a support for single-atom Ni, heralding a promising direction for advanced catalyst design in H_2_O_2_ photosynthesis. Apart from UiO–66–NH_2_ and CdS, the hydrothermal treatment of Red Phosphorus (RP) leads to the formation of P–H and P–OH functional groups on the surface [45]. These groups are integral to the deposition of Ni single-atom species, as they specifically interact with Ni^2+^ ions to create P–Ni and P–O–Ni bonds. Such bonds stabilize Ni atoms and prevent their reduction to neutral Ni^0^, thereby enabling the formation of single-atom Ni sites. The hydrothermal process also imparts an amorphous and mesoporous structure to RP, providing numerous sites for single-atom Ni generation and potentially enhancing reaction efficiency. Techniques such as XANES and EXAFS confirm this formation, with the unique bonding of Ni with RP contributing to specific reactivity that supports H_2_O_2_ synthesis via a 2e^−^ pathway. The presence of two distinct oxidation states of Ni further bolsters performance, providing diverse reaction pathways. Single-atom Ni sites inhibit radiative electron–hole recombination, expedite the photocatalytic process, and elevate overall efficiency. Despite potential corrosion concerns with H_2_O_2_, the Ni-HRP system's optimal H_2_O_2_ production at 30 °C, and the prospect of using a fixed-bed reactor, reinforce stability.

In conclusion, the development and optimization of support materials for SACs have proven to be paramount in the advancement of photocatalytic H_2_O_2_ production. This research showcases a multitude of promising support materials including graphitic carbon nitride, cadmium sulfide, UiO–66–NH_2_, and red phosphorus, each offering distinct advantages in stabilizing single atoms and enhancing catalytic activity. The intricate interplay of the properties such as the electronic environment, structural confinement, and strong metal-support interaction has been instrumental in tailoring the performance of single-atom catalysts. Among the examined supports, g-C_3_N_4_ stands out due to its cost-effective synthesis, rich nitrogen chemistry, and high thermal stability, which collectively make it an attractive choice for SACs. Innovations in support material design are opening up new pathways for enhanced catalytic activity, selectivity, and stability. These discoveries are not only contributing to the effective production of hydrogen peroxide but also laying the groundwork for further exploration and exploitation of SACs in various catalytic processes.

#### Coordination Environment

**Fig. 3:**
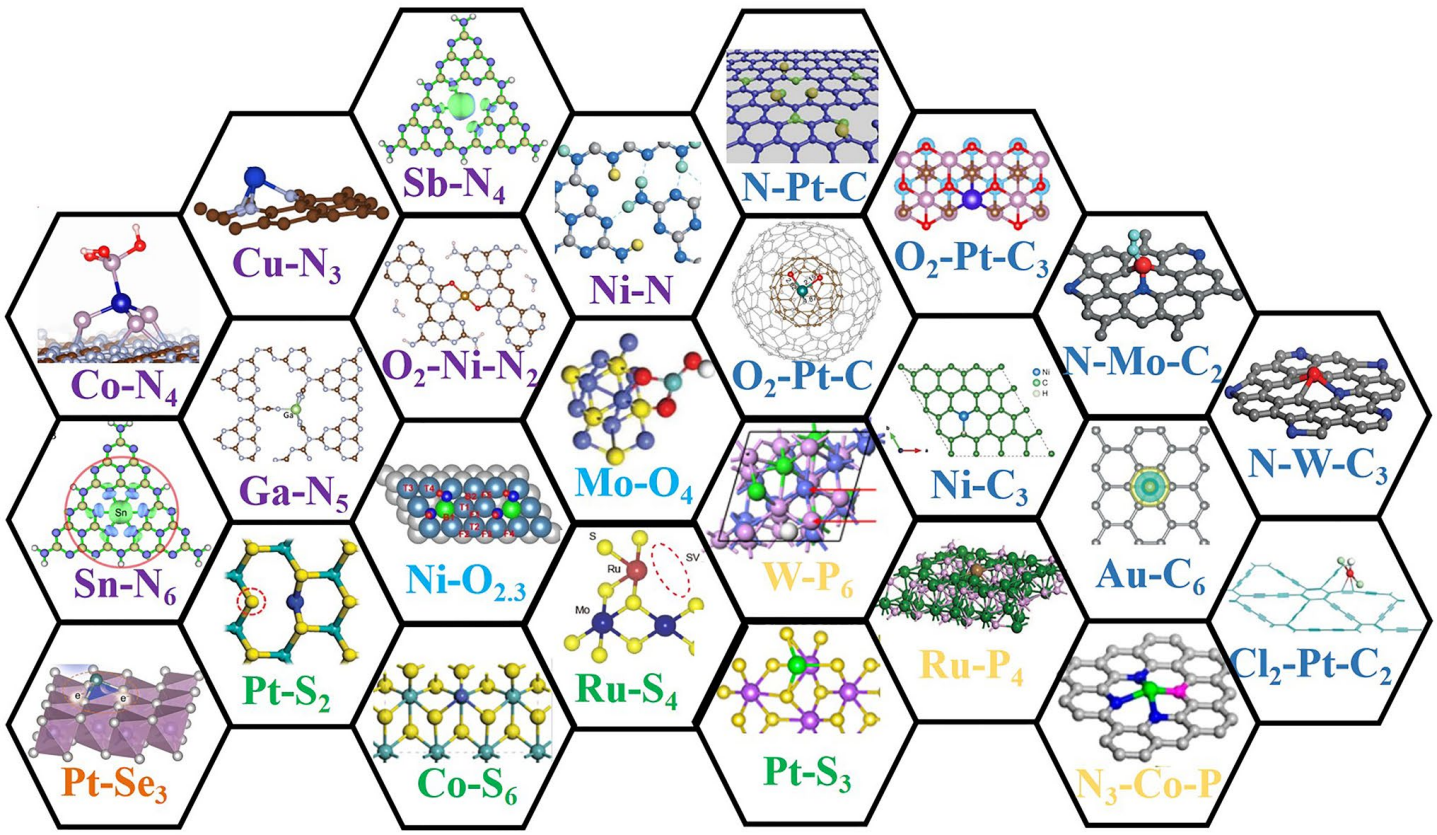
The active moieties of various single atom catalysts. [150] Copyright 2015, Royal Society of Chemistry; [151] Copyright 2019, Royal Society of Chemistry; [103, 152] Copyright 2021, Royal Society of Chemistry; [117] Copyright 2021, Elsevier; [153] Copyright 2015, Springer Nature; [154] Copyright 2016, Springer Nature; [155] Copyright 2018, Springer Nature; [156–158] Copyright 2019, Springer Nature; [159, 160] Copyright 2021, Springer Nature; [161] Copyright 2015, Wiley-VCH; [162, 163] Copyright 2017, Wiley-VCH; [164–166] Copyright 2018, Wiley-VCH; [167] Copyright 2019, Wiley-VCH; [168] Copyright 2020, Wiley-VCH; [169] Copyright 2019, American Chemical Society; [170, 171] Copyright 2020, American Chemical Society; [172] Copyright 2020, American Association for the Advancement of Science

The coordination environment of a single atom catalyst is defined by the nature and configuration of surrounding atoms or ligands that encircle the central metal atom [74]. This environment is pivotal in influencing the catalytic performance of single atom catalysts. Specifically, key descriptors of the coordination environment for single atom catalysts encompass the kinds of atoms bonded to the central atom, their number (coordination number), and the spatial arrangement of these bonds [75]. Typically, single metal atoms find themselves enveloped by elements such as Nitrogen (N) [76], Oxygen (O) [77], Sulfur (S) [78], Carbon (C) [79, 80], phosphorus [45], Selenium (Se), which all play integral roles in the metal atom's stability and reactivity (Fig. 3). In the domain of H_2_O_2_ photosynthesis, particular emphasis is placed on nitrogen, sulfur, and phosphorus as indicated in Table 1.

*Type of Surrounding Atoms* The surrounding atoms in SACs is to stabilize the single atom, preventing it from aggregating with other atoms, which could diminish its unique catalytic properties [37, 81]. Additionally, surrounding atoms can alter the electronic properties of the single atom, thereby influencing its reactivity. Moreover, the support or surrounding atoms can influence how reactant molecules approach and interact with the single atom. They can potentially aid in the diffusion of reactants to the active site or the removal of products, ensuring efficient catalytic turnover. Thus, the selection of support or surrounding atoms is crucial in SACs, with extensive research dedicated to identifying the best combinations for particular reactions. Materials like g-C_3_N_4_, CdS, and red phosphorus are among the most studied supports for single-atom catalysis in H_2_O_2_ photosynthesis.

Predominantly, Nitrogen stands out as the chief coordinating atom. This prominence stems from the three σ bonds engaged by each Nitrogen atom in g-C_3_N_4_, leaving a singular lone electron pair to interact with the metal atom [14, 82–84]. As a result, each Nitrogen atom can coordinate with only one metal atom, leading to the formation of single atom catalysts. In these catalysts, the M–N coordination plays a pivotal role in ensuring the dispersion and stability of single atoms and influencing their electronic structure and reactivity. Moreover, in the synthesis of Pt/CdS, the selective bonding between single atom platinum (Pt) and sulfur (S) manifests as a consequence of several interrelated factors. Primarily, the unoccupied d-orbitals of Pt atoms present a unique opportunity to establish strong coordination bonds with the filled p-orbitals of sulfur. This interaction is facilitated by the availability of sulfur's lone pair electrons, resulting in a robust and stable bonding arrangement. Within the CdS lattice structure, the specific orientation and exposure of sulfur atoms may further augment their accessibility to Pt, rendering sulfur sites preferentially available for reaction. Moreover, the formation of Pt–S bonds exhibits a potential thermodynamic advantage, culminating in a more stable final product, and thereby underlining the preferential nature of this reaction pathway. From a kinetic perspective, lower energy barriers to Pt–S bond formation may favor the reaction with sulfur, enhancing its feasibility relative to alternative reactions. The interaction between Pt and S is also influenced by Pt's inherent Lewis acidic character and sulfur's Lewis basicity, a complementary relationship that encourages the formation of the bond. Furthermore, in the intricate synthesis of single atom Nickel (Ni) on red phosphorus, the formation of a Ni–P bond manifests through a multifaceted interplay of electronic and structural considerations. The partially filled 3*d* orbital of Ni holds the potential to overlap with the 3*p* orbital of phosphorus (P), thereby enabling the creation of a robust covalent or dative bond. This bonding capacity is further accentuated by the interaction between Ni's unoccupied *d*-orbitals and the lone pair electrons resident in red phosphorus, culminating in the Ni–P bond formation. Within the supportive matrix of red phosphorus, the surface structure and inherent defects become pivotal to the bonding mechanism, potentially exposing specific P sites amenable to favourable interactions with Ni atoms. The affinity between Ni and P results not just from spatial alignment but is also deeply connected to red phosphorus's distinct chemical characteristics. Factors such as oxidation states, coordination environments, and other chemical properties synergistically contribute to this affinity, thereby amplifying the formation of Ni–P bonds. Collectively, these elements elucidate the complex mechanism governing the Ni–P bond formation, providing valuable insights into potential applications and functionalities of single atom Ni supported on red phosphorus.

*Coordination Number* This is the number of atoms or ligands that a single atom is directly bonded to. The coordination number can greatly impact the reactivity and selectivity of the catalyst as it influences the geometry of the bonds and thus the electronic structure of the single atom [85–87]. From the perspective of metal-nitrogen coordination in g-C_3_N_4_, the number of metal-nitrogen coordination can significantly influence the electronic structure and catalytic performance [88, 89]. This stems from nitrogen's high electronegativity, which can alter the charge distribution around the metal atom, modulating its interaction with reactants [90, 91]. For instance, the number of metal-nitrogen bonds can impact the electron density around the metal atom. This could potentially enhance the catalytic performance by promoting adsorption of reactants or facilitating electron transfer [92, 93]. However, it's also worth noting that too many metal-nitrogen bonds might lead to a strong electron-withdrawing effect, which could negatively impact the catalytic performance by hindering the release of products [94–96]. Hence, it's essential to strike a balance when it comes to the number of metal-nitrogen bonds in the coordination environment. In summary, the number of metal-nitrogen coordination in single atom catalysts of carbon nitride can significantly impact the catalytic properties. However, the exact impact can depend on various factors, including the type of metal atom, the specific catalytic process, and the overall coordination environment. Therefore, careful design and optimization of the catalyst are crucial for achieving the desired catalytic performance.

*Bond Geometry* The bond geometry, or the spatial arrangement of bonds around the metal atom, is another key aspect of the coordination environment [97]. It is directly related to the coordination number and also has a significant influence on the electronic structure of the single atom, thereby affecting the catalytic performance. In single atom catalysts, the bond geometry can be diverse, including trigonal planar, tetrahedral, square planar, and octahedral, among others [98–102]. From Table 1, it can be observed that most single atom catalysts on carbon nitride support have M–N_3_ or M–N_4_ coordination geometries, suggesting the prevalence of these bond geometries in this type of catalysts [28]. This is because the structure of graphitic carbon nitride is composed of tri-*s*-triazine units, which are nitrogen-rich and connected by amino groups. In g-C_3_N_4_, every nitrogen atom possesses a lone pair of electrons, facilitating its bond formation with the metal atom, thus stabilizing it as a single atom catalyst [53]. This structural characteristic provides an ideal environment for M–N_3_ or M–N_4_ coordination geometries, which can ensure the stability of the single atom by forming strong bonds with nitrogen atoms, maintain the dispersion of the single atoms by preventing them from agglomerating, and enhance the reactivity by modifying the band structure and electronic properties. For example, Li et al. investigate the intricate effects of coordination by incorporating single-atom nickel (Ni) into the porous coordination network (PCN) framework, resulting in discernible alterations in the electronic structure [103]. This calculated modification instigates an expansion in the optical absorption spectrum and refines the charge transfer processes. Specifically, the optical absorption redshift, induced by this alteration, broadens the wavelength response into the visible light region, an essential feature for photocatalytic applications. The manifestation of two distinct band gaps at 2.73 and 2.3 eV, respectively ascribed to conventional band-to-band transitions in PCN and the unique Ni–N coordination structure, enables a metal-to-ligand charge transfer (MLCT) process. Concurrently, the formation of novel LUMO states through the hybridization of Ni's 4*s* orbitals with nitrogen's 2*p* orbitals results in a narrowed band gap of 2.3 eV, thereby facilitating efficient electron transitions. This adjustment is instrumental in driving both water reduction and oxidation reactions, underpinning the enhancement of photocatalytic reactions. Furthermore, the judicious integration of Ni leads to the sustained generation of H_2_ from pure water, all the while extending optical absorption and diminishing charge carrier recombination, as evidenced by a decrease in photoluminescence emission. Collectively, these synergistic effects provide a remarkable boost in photocatalytic performance for overall water splitting, thereby illuminating a novel avenue towards efficient solar-to-fuel conversion.

In summary, the coordination environment of a single atom catalyst profoundly influences its electronic structure and, consequently, the reactivity of the single atom. Through meticulous adjustment of this environment, one can optimize the catalytic performance of single atom catalysts. This is one of the major advantages of single atom catalysts over traditional bulk catalysts, as it allows for atomic-level control and customization of the catalyst. The coordination environment is also directly influenced by the host material. Different support materials can provide different types of coordination sites for the single atoms, allowing for a wide range of possible coordination environments. This further underscores the importance of the support material choice in the design of single atom catalysts. The careful design of the coordination environment and support material allows for the customization of single atom catalysts for specific catalytic reactions, making them a powerful tool in photocatalysis.

### Synthesis Method of Single Atom Catalysts

Single atom catalysts have emerged as a pioneering area within catalytic research, distinguished by their unmatched efficiency and unique reactivity profiles. These characteristics set them apart from conventional nanoparticulate catalysts. As the push for more sustainable and efficient pathways for H_2_O_2_ photosynthesis grows, there is a pressing need to deepen our understanding of SACs synthesis methods and fine-tune them for this specific application [104]. A spectrum of synthesis approaches, from the pyrolysis method to impregnation method, has been investigated to adeptly anchor solitary metal atoms onto diverse supports. This anchors not only offer stability and dispersion but also potentiate their catalytic prowess. In this section, we will comprehensively discuss the synthesis techniques for SACs, with a special emphasis on tailoring and optimizing these methods to address the unique challenges posed by H_2_O_2_ photosynthesis.

#### Pyrolysis Method

**Fig. 4:**
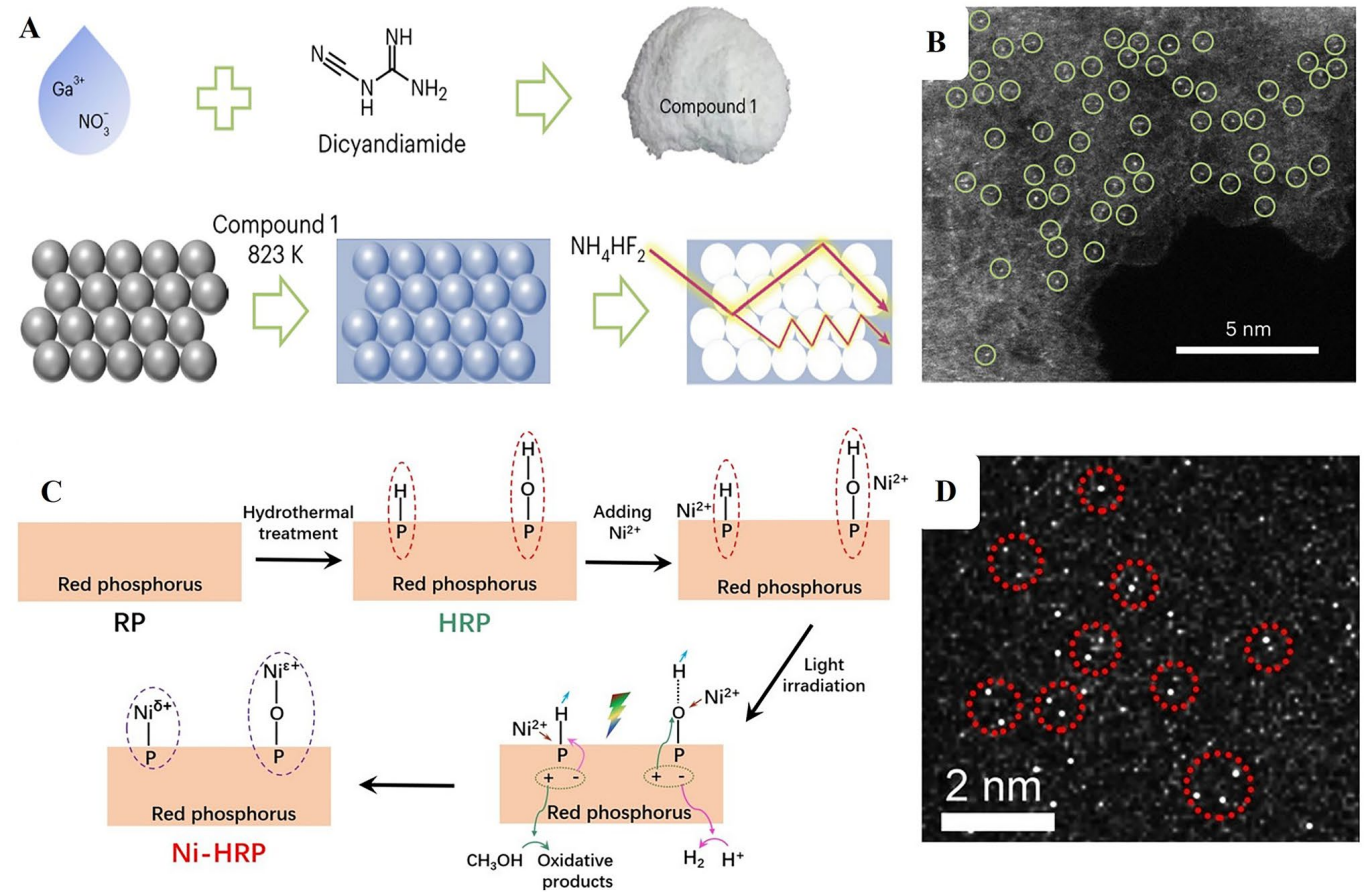
**A** Synthetic route for Ga–N_5_ atomic site on macroporous inverse-opal-type carbon nitride (CNIO-GaSA). **B** HAADF-STEM image of CNIO-GaSA. Reproduced with permission [41], Copyright 2023, Springer Nature. **C** Illustration of reactive-group guided synthesis of single atom Ni- hydrothermally treated red phosphorus (Ni-HRP), HAADF-STEM image of Ni-HRP. Reproduced with permission [45], Copyright 2022, John Wiley & Sons, Inc

Single atom catalysts have rapidly ascended in prominence, primarily due to their unique electronic attributes and the maximized exploitation of metal-active sites. In the cohort of possible supports for SACs, g-C_3_N_4_ is particularly noteworthy. The synthesis of SACs on g-C_3_N_4_ through pyrolysis, specifically optimized for H_2_O_2_ photosynthesis, constitutes a multi-faceted procedure [48]. This process strategically aims to anchor distinct metal atoms onto the g-C_3_N_4_ scaffold to leverage their unparalleled catalytic characteristics. Initially, the selection of metal precursors, varying from metal salts to metal–organic compounds, is paramount, influencing the subsequent anchoring and dispersion of the individual metal atoms. Concurrently, g-C_3_N_4_ is derived via pyrolysis of specific precursors like melamine or urea [105]. In an oxygen-free, high-temperature environment, these substances undergo thermal decomposition, resulting in the signature layered g-C_3_N_4_ structure with its distinctive electronic properties. Following this step, the designated metal precursor is amalgamated with the g-C_3_N_4_ substrate, which pre-emptively facilitates the anchoring of metal atoms onto the g-C_3_N_4_ structure during pyrolysis [26]. Exposing this combination to pyrolysis, typically within an inert ambience like nitrogen or argon, the escalating temperature instigates metal precursor decomposition, culminating in ideally anchored individual metal atoms on the g-C_3_N_4_ base (Fig. 4A–B) [41]. The meticulous modulation of pyrolysis parameters (spanning temperature, duration, and atmosphere) becomes vital, guiding the eventual spatial distribution of metal atoms. Such precision ensures isolated anchoring, optimizing catalytic prowess while inhibiting metal aggregate formation. The final product is a structured SACs/g-C_3_N_4_ composite with single, isolated metal atoms that serve as catalytic epicenters for H_2_O_2_ photosynthesis. Depending on the application, post-synthesis treatments might be necessitated to fine-tune the catalyst's oxidation state or to augment its stability. At its core, this synthesis mechanism hinges on the controlled thermal degradation of precursors, crafting a specialized SACs/g-C_3_N_4_ configuration primed for H_2_O_2_ photosynthesis efficiency, and epitomizing the harnessing of singular metal atom catalytic properties.

#### Impregnation Method

The impregnation method in the synthesis of single atom catalysts refers to a process in which metal precursors are uniformly dispersed onto a support material, with the goal of anchoring individual metal atoms onto the support in their isolated form, rather than as nanoparticles or clusters [26]. The synthesis of SACs on g-C_3_N_4_ support using the impregnation method, specifically tailored for H_2_O_2_ photosynthesis, commences with the preparation of the g-C_3_N_4_ substrate through the thermal polymerization of precursors such as melamine or urea. This results in a layered g-C_3_N_4_ structure characterized by its distinct electronic properties and a favorable band gap for photocatalytic processes [27]. The subsequent selection of an apt metal precursor, which may vary from metal salts to metal–organic compounds, is crucial, given its determinative role in shaping the final catalytic attributes of the SACs. During the impregnation step, the g-C_3_N_4_ support encounters a solution of the chosen metal precursor, ensuring its adherence to the g-C_3_N_4_ surface as the solvent evaporates. This is followed by a controlled thermal treatment, aimed at decomposing the metal precursor and facilitating the deposition of single, isolated metal atoms onto the g-C_3_N_4_ matrix. The conditions of this thermal process are meticulously adjusted to maintain the isolated dispersion of metal atoms, preventing the emergence of larger aggregations [106]. A potential post-treatment activation phase ensures that the metal atoms are in the requisite oxidation state, enhancing their catalytic prowess. In summary, the impregnation synthesis of SACs/g-C_3_N_4_ is a methodical progression of steps aimed at harnessing the optimal potential of g-C_3_N_4_ supported SACs for H_2_O_2_ photosynthesis.

#### Photoreduction Method

The photoreduction method stands out as a pioneering technique in the synthesis of SACs, particularly with the incorporation of g-C_3_N_4_ as the foundational support. Central to this approach is the inherent photocatalytic prowess of g-C_3_N_4_ underpinned by its optimal band gap and structured electronic profile [48]. Upon illumination, it spawns electron–hole pairs, serving as key players in reduction reactions. This photo-excitation facilitates a streamlined reduction of metal precursors, predominantly metal cations, anchoring them as isolated atoms onto the g-C_3_N_4_ matrix. The process boasts precision, allowing fine-tuned deposition control by adjusting parameters such as light intensity, wavelength, and exposure duration. This ensures the creation of SACs without aggregation. Operating under ambient conditions, photoreduction ensures the integrity of both g-C_3_N_4_ and metal precursors, and avoids undesirable alterations [45]. A symbiotic relationship between the photo-activated g-C_3_N_4_ and metal precursors augments the stability of SACs. The technique's adaptability is evident from its compatibility with a spectrum of metal precursors, ranging from rudimentary salts to intricate metal–organic compounds. Moreover, being reliant on light energy, the method treads an eco-friendly path [73]. The intrinsic characteristics of g-C_3_N_4_ can be tailored to bolster its synergy with specific metal precursors or to amplify its photocatalytic fervor. To encapsulate, the photoreduction method, with g-C_3_N_4_ as its backbone, presents a refined, eco-conscious, and versatile blueprint for SACs synthesis. Furthermore, the surface of red phosphorus (RP) is hydrothermally treated to introduce P–H and P–OH groups, which act as pivotal anchoring sites for Ni ions [45]. Upon dispersing this treated RP in a Ni^2+^ methanol aqueous solution and subsequent light exposure, photoelectrons (e^−^) and holes (h^+^) are generated, setting the stage for the precise deposition of Ni (Fig. 4C–D). Specifically, near P–H groups, Ni^2+^ ions capture the photoelectrons, giving rise to Ni^δ+^ species, where δ < 2. Concurrently, a P–Ni bond forms as hydrogen is substituted, preventing Ni from reducing to its neutral state and ensuring its anchorage to RP. Meanwhile, the resultant holes oxidize methanol. In the context of P–OH groups, the hydrogen ionizes, leaving P–O- which bonds with Ni^2+^ to form P–O–Ni. Here, holes serve to oxidize the Ni to Ni^ϵ+^ with ϵ > 2, while earlier-generated photoelectrons neutralize the surrounding H^+^ ions. Remarkably, the specificity of these reactive sites and their respective conditions favor the formation of single-atom Ni sites over nanoparticles, emphasizing the method's precision. This is further validated by X-ray photoelectron spectroscopy (XPS), a robust technique elucidating elemental composition and chemical states. XPS results are in harmony with the described reactions, unequivocally demonstrating the formation of single-atom Ni sites on RP. The ingenuity of this deposition design lies in its ability to preferentially create single-atom sites, offering valuable insights for the future design and synthesis of advanced single-atom catalysts.

### Characterization Methods of Single Atom Catalysts

#### Scanning Transmission Electron Microscopy (STEM)

**Fig. 5:**
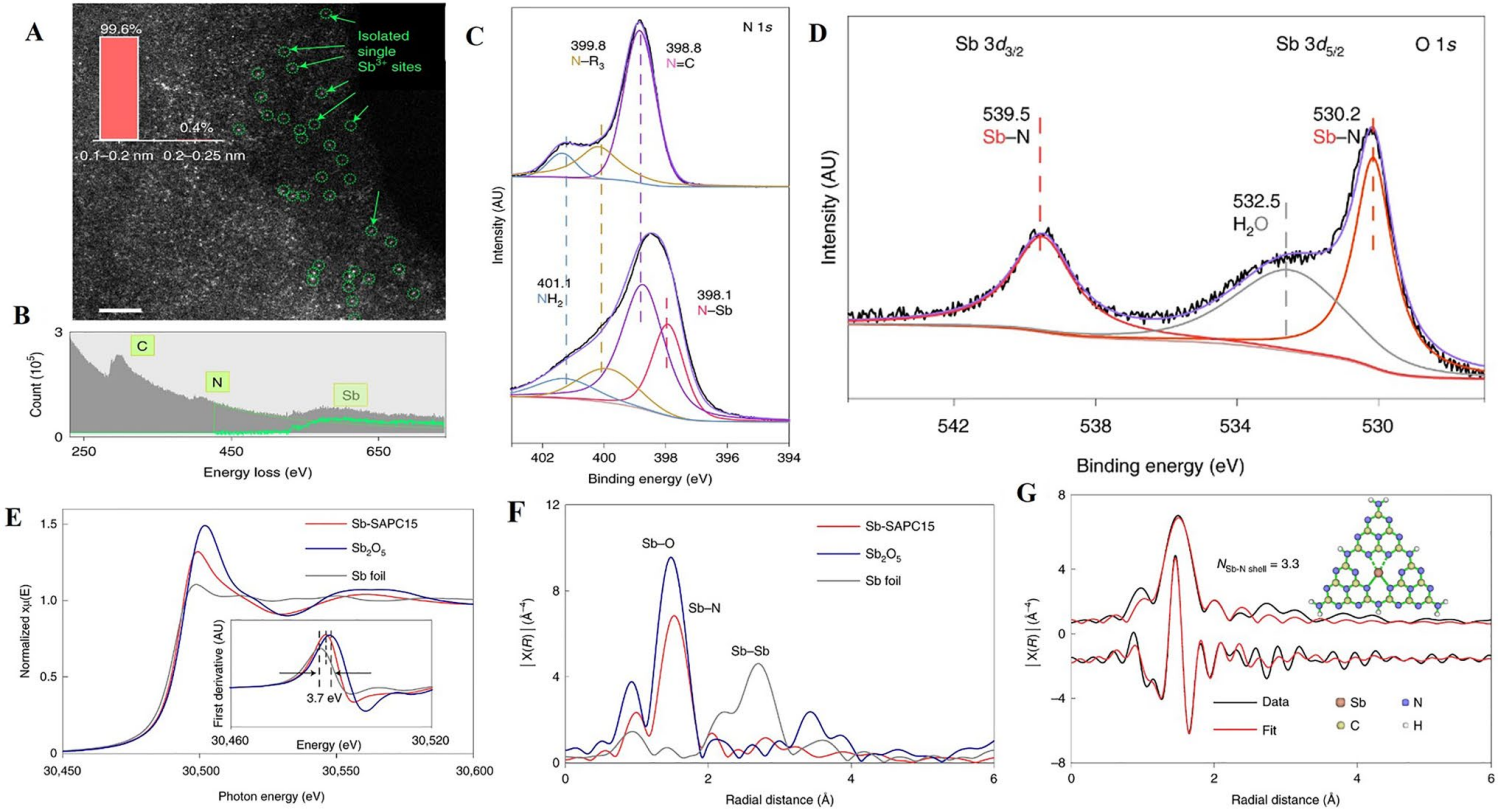
**A** High-magnification HAADF–STEM image of Sb-SAPC15. The inset is the size distribution of the bright spots. Scale bar, 2 nm. **B** EELS spectrum of Sb-SAPC15. **C** High-resolution N 1*s* XPS spectra of PCN (up) and Sb-SAPC15 (down) and Sb 3d. **D** XPS spectrum of Sb-SAPC15. **E** Sb K-edge X-ray absorption near edge structure and **F** Fourier transform–EXAFS spectra of the Sb foil, Sb_2_O_5_ and Sb-SAPC15. **G** Fitting of the EXAFS data of the Sb-SAPC15 based on the model obtained from DFT optimization. The insets show optimized molecular models based on DFT for EXAFS fitting. R indicates the radial distance in Å. Reproduced with permission [107], Copyright 2021, Springer Nature

STEM represents a pivotal tool in the modern characterization of SACs [24]. Owing to its atomic-level resolution, STEM facilitates the direct observation and examination of individual atoms within a given catalyst structure. One highly significant application of STEM in the context of SACs is the use of High-angle annular dark-field (HAADF) imaging. This specialized technique is specifically tailored to detect the contrast between heavy metal atoms and the typically lighter support materials. Consequently, HAADF imaging within the STEM framework as well as Electron Energy Loss Spectroscopy (EELS) can offer unparalleled insights into the spatial distribution and environment of single atoms within the catalyst (Fig. 5A–B) [107]. This understanding is critical in elucidating the unique properties and functional behaviors of SACs, paving the way for innovative applications in various catalytic processes [53]. By bridging the gap between the macroscopic properties and atomic-level structure, STEM and its associated methodologies continue to provide a vital window into the complex world of single atom catalysis.

#### X-ray Absorption Spectroscopy (XAS)

XAS, comprising two principal techniques, namely X-ray absorption near-edge structure (XANES) and extended X-ray absorption fine structure (EXAFS), serves as an indispensable method in the characterization of SACs [24, 107]. These complementary techniques provide nuanced insights into the electronic structure and local atomic configuration of SACs. XANES, with its sensitivity to subtle changes in the oxidation state, facilitates precise determinations of the electronic structure of individual atoms, allowing researchers to explore the correlation between oxidation state and catalytic behavior. On the other hand, EXAFS delivers detailed information about the local atomic structure surrounding the absorbing atom. It offers a comprehensive understanding of the coordination environment, bond lengths, and angular relationships between neighboring atoms (Fig. 5E–G) [107, 108]. Together, XANES and EXAFS extend the characterization toolbox for SACs, promoting a holistic view of their fundamental properties and behaviors. This enhanced understanding contributes significantly to the rational design and optimization of SACs, with far-reaching implications for their application in diverse catalytic processes.

#### X-ray Photoelectron Spectroscopy

XPS represents a vital analytical technique in the study of SACs, offering unique insights into the complex interplay between surface composition and electronic structure [58]. By probing the kinetic energy of emitted photoelectrons in response to X-ray irradiation, XPS allows for the precise determination of both the elemental composition and oxidation states present at the surface of SACs. This specificity renders XPS an indispensable tool for researchers investigating the dynamic behavior of individual atoms within SACs. Its capacity to elucidate the intricate relationship between oxidation state and catalytic activity lends critical support to the targeted design and optimization of SACs. Furthermore, the surface sensitivity of XPS promotes a deeper understanding of the interaction between the isolated metal atoms and the supporting substrate (Fig. 5C–D) [107, 109]. In conjunction with other characterization techniques, XPS continues to contribute significantly to the rapidly expanding field of single atom catalysis, enabling scientific advances with potential applications across various industrial and environmental domains.

#### Nuclear Magnetic Resonance (NMR) Spectroscopy

NMR spectroscopy emerges as a powerful analytical method in the study of SACs, offering unparalleled insights into the local environment of specific nuclei [110]. Unlike traditional surface-sensitive techniques, NMR delves into the coordination sphere of single atoms, providing valuable data about their electronic environment and spatial arrangement within the support matrix. By exploiting the magnetic properties of nuclei and their interaction with an applied magnetic field, NMR reveals critical information regarding the electronic structure, chemical bonding, and connectivity of the individual atoms within SACs. This ability to probe the local environment helps in identifying unique features, such as coordination numbers and ligand types, that define the behavior of single atoms in catalytic processes. Moreover, NMR's sensitivity to subtle changes in the local electronic environment enables researchers to track dynamic processes, such as changes in oxidation state or structural rearrangements during catalysis. In concert with complementary techniques, NMR Spectroscopy contributes significantly to the comprehensive characterization of SACs, paving the way for a deeper understanding of their structure–function relationships, and thereby aiding in the rational design of more efficient and selective catalysts.

#### Computational Methods

Computational Methods, encompassing techniques such as DFT, have emerged as invaluable tools in the exploration and understanding of SACs [57]. Unlike experimental techniques, computational methodologies offer the unique ability to probe the intrinsic characteristics of SACs at the atomic and electronic levels. DFT, in particular, has proven instrumental in modeling the complex interactions within SACs, enabling detailed insights into their stability, electronic structure, and reactivity. By solving the quantum mechanical equations governing the behavior of electrons, DFT facilitates the prediction of various properties and behaviors of single atoms in different coordination environments. This theoretical framework, in conjunction with experimental validation, allows for the systematic exploration of the underlying principles that guide the function of SACs. Furthermore, the computational investigation of SACs supports the rational design of catalysts by predicting optimal structural and electronic configurations for specific catalytic processes. As SACs continue to gain prominence in both academic research and industrial applications, the synergy between computational methods and experimental techniques is poised to drive forward the frontiers of our understanding and utilization of these fascinating materials.

### Reaction Paths of Photocatalytic H_2_O_2_ Production over Single Atom Catalysts

**Fig. 6:**
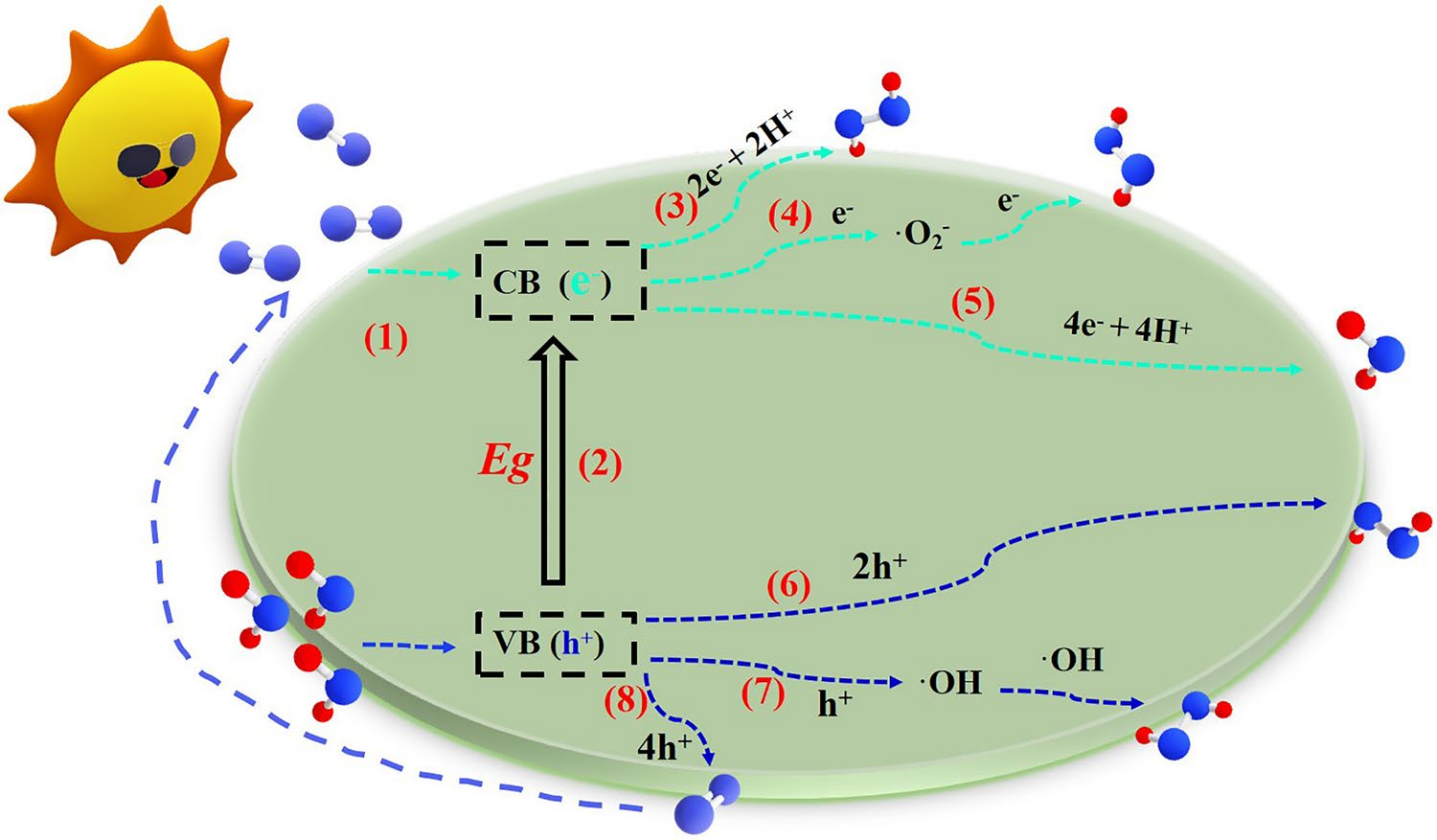
Schematic diagram of H_2_O_2_ photosynthesis: (1) light harvesting; (2) charge excitation; (3) One-step two-electron route of oxygen reduction reaction; (4) Two-step one-electron route of oxygen reduction reaction; (5) Four-electron route of oxygen reduction reaction; (6) Two-electron pathway of water oxidation reaction; (7) One-electron pathway of water oxidation reaction; (8) Four-electron pathway of water oxidation reaction

The photocatalytic generation of H_2_O_2_ involves the interaction of light with a semiconductor photocatalyst, which causes excitation of electrons from the valence band (VB) to the conduction band (CB), leaving holes (h^+^) in the VB [111]. These photogenerated electrons and holes can interact with water and oxygen molecules, and through a series of reactions, produce hydrogen peroxide (Fig. 6). There are generally two pathways through which this can occur: oxygen reduction reaction (ORR) and water oxidation reaction (WOR) [112, 113].

#### Oxygen Reduction Reaction

In this route, oxygen in the reaction medium can be reduced to form hydrogen peroxide. There are three potential ORR pathways.

**One-step two-electron route** In this pathway, oxygen directly interacts with two electrons and two protons to form hydrogen peroxide. The typical representation of the reaction is as follows:

1 $${\text{O}}_{{2}} + {\text{ 2e}}^{ - } + {\text{ 2H}}^{ + } \to {\text{H}}_{{2}} {\text{O}}_{{2}} \quad + 0.{\text{68V versus NHE}}$$

**Two-step single-electron route** Here, oxygen first interacts with a single electron to form superoxide (·O_2_^−^), and then the superoxide reacts with a second electron and two protons to form hydrogen peroxide (H_2_O_2_) [114]. The reaction is generally represented as follows:

2 $${\text{O}}_{{2}} + {\text{ e}}^{ - } \to {\text{O}}_{{2}}^{ - } \quad - 0.{\text{33V versus NHE}}$$

3 $${\text{O}}_{{2}}^{ - } + {\text{ e}}^{ - } + {\text{ 2H}}^{ + } \to {\text{H}}_{{2}} {\text{O}}_{{2}} \quad + {1}.{\text{44V versus NHE}}$$

**Four-electron ORR competition reaction** This is a competitive pathway where oxygen interacts with four electrons and four protons to form water (H_2_O) instead of hydrogen peroxide [115]. The typical representation of the reaction is as follows:

4 $${\text{O}}_{{2}} + {\text{ 4e}}^{ - } + {\text{ 4H}}^{ + } \to {\text{2H}}_{{2}} {\text{O}}\quad + {1}.{\text{23V versus NHE}}$$

#### Water Oxidation Reaction

In this pathway, water molecules are oxidized to form hydrogen peroxide. Again, there are multiple potential reactions.

**Two-electron pathway** Here, two water molecules interact with two holes to produce hydrogen peroxide and two protons. The typical representation of the reaction is usually depicted as follows:

5 $${\text{2H}}_{{2}} {\text{O }} + {\text{ 2h}}^{ + } \to {\text{H}}_{{2}} {\text{O}}_{{2}} + {\text{ 2H}}^{ + } \quad + {1}.{\text{76V versus NHE}}$$

**Single-electron WOR reaction** This reaction produces hydroxyl groups, which can recombine to form hydrogen peroxide. The usual representation of the reaction is typically as follows:

6 $${\text{H}}_{{2}} {\text{O }} + {\text{ h}}^{ + } \to {\text{OH }} + {\text{ H}}^{ + } \quad + {2}.{\text{73V versus NHE}}$$

7 $${\text{OH }} + {\text{OH}} \to {\text{H}}_{{2}} {\text{O}}_{{2}}$$

**Four-electron reaction** This is a competitive pathway where two water molecules interact with four holes to produce oxygen and four protons instead of hydrogen peroxide. The typical representation of the reaction is usually expressed in the following manner:

8 $${\text{2H}}_{{2}} {\text{O }} + {\text{ 4h}}^{ + } \to {\text{O}}_{{2}} + {\text{ 4H}}^{ + } \quad + {1}.{\text{23V versus NHE}}$$

The exact pathway that occurs depends on the specific photocatalyst used and the reaction conditions, such as light intensity and wavelength, temperature, and pH. It's also important to note that the efficiency of H_2_O_2_ production can be hindered by several factors, including charge carrier recombination, poor light harvesting ability, and unfavourable reaction kinetics and thermodynamics. This makes the design and optimization of efficient photocatalysts for H_2_O_2_ production a challenging task.

Single atom catalysts could potentially improve the efficiency of these reactions by providing highly active and selective sites for the reactions to occur, reducing charge carrier recombination, and improving light harvesting ability. However, the exact behaviour would depend on the specific type of single atom catalyst used.

## Roles of Single Atoms in Photocatalytic H_2_O_2_ Production

In photocatalytic H_2_O_2_ production, the role of single atoms has become an increasingly pertinent topic due to the unique properties and precise atomic control they offer. Here, we'll delve deeper into how single atoms enhance light absorption and charge generation, improve the migration and separation of charge carriers, and lower the energy barrier for reactant adsorption and activation.

### Enhancing Light Absorption and Charge Generation

In the realm of photocatalysis, the absorption efficiency of a photocatalyst is crucial. It sets the stage for the process by generating charge carriers, notably electron–hole pairs [116]. Furthermore, the efficiency of subsequent charge generation plays a pivotal role in determining the overall efficacy of photocatalytic reactions.

**Fig. 7:**
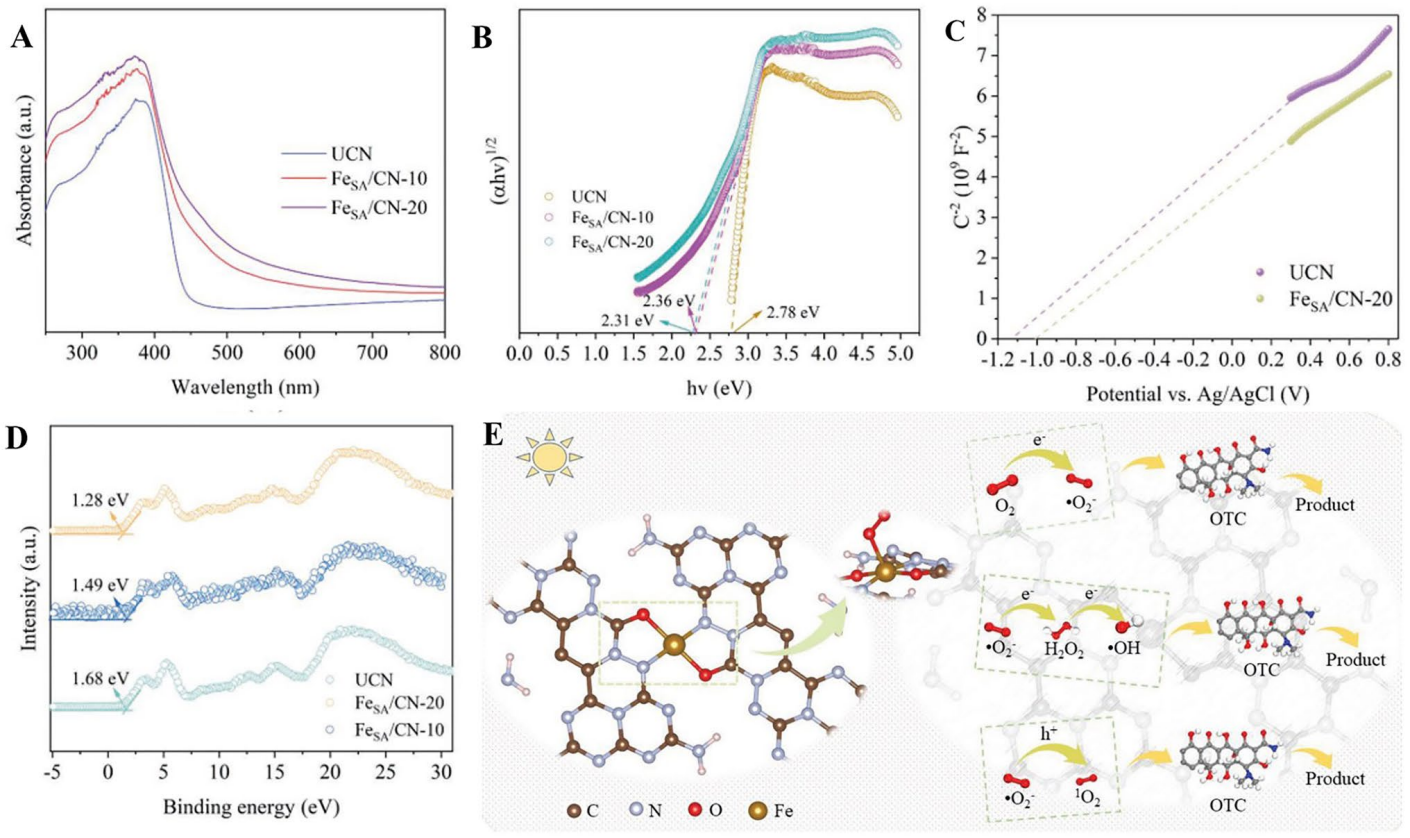
**A** UV–vis DRS spectra of synthesized photocatalyst. **B** Tauc plots of UCN, FeSA/CN-10, and FeSA/CN-20 catalysts. **C** Mott–Schottky plots. **D** VB-XPS spectra of UCN, FeSA/CN-10, **E** FeSA/CN-20; the possible photodegradation mechanism of OTC by FeSA/CN sample. Reproduced with permission [39], Copyright 2023, Wiley- VCH

The band structure of a material, encompassing the valence and conduction bands, is of paramount importance in photocatalysis. It dictates the material's light absorption properties and its capability for efficient charge separation by outlining the electron energy levels within a solid. Illustrating this, Chen’s group pioneered the development of a single Fe atom-modified g-C_3_N_4_ photocatalyst via a pyrolysis method [39]. Their research showcased that the introduction of single Fe atoms induces significant modifications to the bandgap. For context, the pristine g-C_3_N_4_ (UCN) features a bandgap of 2.78 eV, thereby confining its light absorption predominantly to the ultraviolet domain. However, the integration of single Fe atoms diminished this bandgap to 2.31 eV in the FeSA/g-C_3_N_4_ sample. As illustrated in Fig. 7A, UCN exhibits partial visible light absorption with an absorption edge proximate to 450 nm. Upon the incorporation of single Fe atoms, there is a notable enhancement in its light-harvesting capacity. Consequently, FeSA/g-C_3_N_4_ extends beyond mere ultraviolet absorption, encompassing a more expansive range within the visible light spectrum as illustrated in Fig. 7B. Additionally, the Fe atoms introduced notable shifts in the potentials of the conduction and valence bands. Specifically, the UCN's conduction band potential of − 1.32 eV transitioned to − 1.22 eV in the FeSA/g-C_3_N_4_ sample. In parallel, the valence band potential transitioned from 1.68 eV in the UCN sample to 1.28 eV in the FeSA/g-C_3_N_4_ (Fig. 7C). The consequent reduction in the bandgap, coupled with the enlarged light absorption spectrum, synergistically enhances the photocatalytic activity. This improved photocatalyst has an increased propensity to interact with a greater number of photons, thus producing a more substantial quantity of electron–hole pairs, which are fundamental to the photocatalytic mechanism. Moreover, the modified potentials in the conduction and valence bands may enhance charge separation efficiency, reducing the likelihood of recombination events, and thereby amplifying the overall photocatalytic performance.

**Fig. 8:**
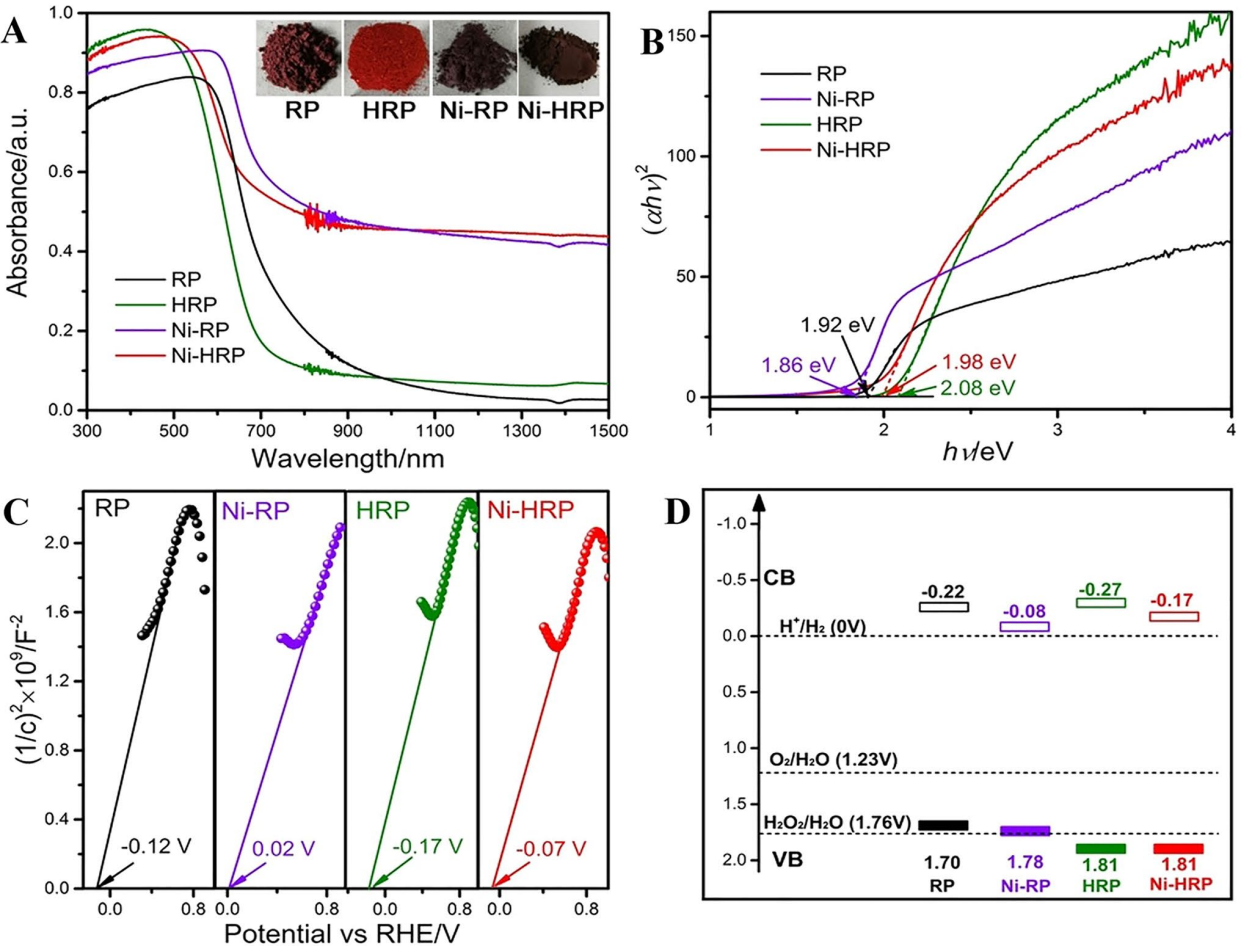
**A** UV/Vis–NIR DRS spectra of prepared samples. Insets are the digital photographs of the photocatalyst powders. **B** Tauc plots, **C** Mott–Schottky plots, and **D** band structure illustration of all samples. Reproduced with permission [45], Copyright 2022, Elsevier

Furthermore, Wang et al. use single atom Nickel to improve the light adsorption of red phosphorus. As shown in Fig. 8A, the incorporation of Ni species significantly enhanced absorption in the visible-infrared spectrum [45]. Upon examining the Tauc plots, we determined the optical band gap values (E_g_) for RP, HRP, Ni-RP, and Ni-HRP to be 1.92, 2.08, 1.86, and 1.98 eV, respectively, as shown in Fig. 8B. The hydrothermal process yielded a slight increase in the band gap for red phosphorus. In contrast, introducing Ni-based cocatalysts led to a reduction in this gap. The Mott–Schottky plots, displayed in Fig. 8C, provided insights into the flat band potentials of all samples. Generally, the conduction band (CB) edge of n-type semiconductors is observed to be approximately 0.1 V more negative than the flat band potential. As such, the CB positions of both HRP and Ni-HRP were identified. Figure 8D illustrates the band structures of HRP and Ni-HRP. Analysis of the energy band reveals that the positions of CB and VB for both HRP and Ni-HRP align well with the requirements for efficient water splitting.

In summary, by modifying the band structure and electronic properties of photocatalysts, single atom catalysts can enhance both light absorption and charge generation. This unique capability makes single atom catalysts a highly promising tool for optimizing photocatalytic reactions. Future research may focus on developing innovative methods to design and control single atom catalysts to further enhance their impact on band structure and electronic property modification, improving the photocatalytic efficiency in H_2_O_2_ production.

### Improving the Migration and Separation of Charge Carriers

The efficiency of photocatalysis relies significantly on the efficient generation and migration of charge carriers (e.g., electrons and holes). When a photocatalyst absorbs light, electron–hole pairs are generated. The separation and transport of these charge carriers to the catalyst's surface are necessary to interact with the reactants, leading to the desired chemical transformation. If these charge carriers recombine before reaching the reaction site, no chemical reaction occurs, decreasing the efficiency of the photocatalysis process. Besides, the charge carriers should be able to efficiently transfer to the reactants in a photocatalytic reaction. Improving the migration of charge carriers could increase the chance of them reaching the reaction sites and transfer their charges to the reactants. This will result in a higher reaction rate and enhanced photocatalytic performance. In the case of single atom catalysts, the unique electronic structure allows for more efficient generation and separation of charge carriers compared to their bulk counterparts. Moreover, the interaction between the single atom and the substrate, including charge transfer, redistribution of electron density, and alteration of energy levels, can further enhance the separation and migration of charge carriers, leading to improved photocatalytic performance.

**Fig. 9:**
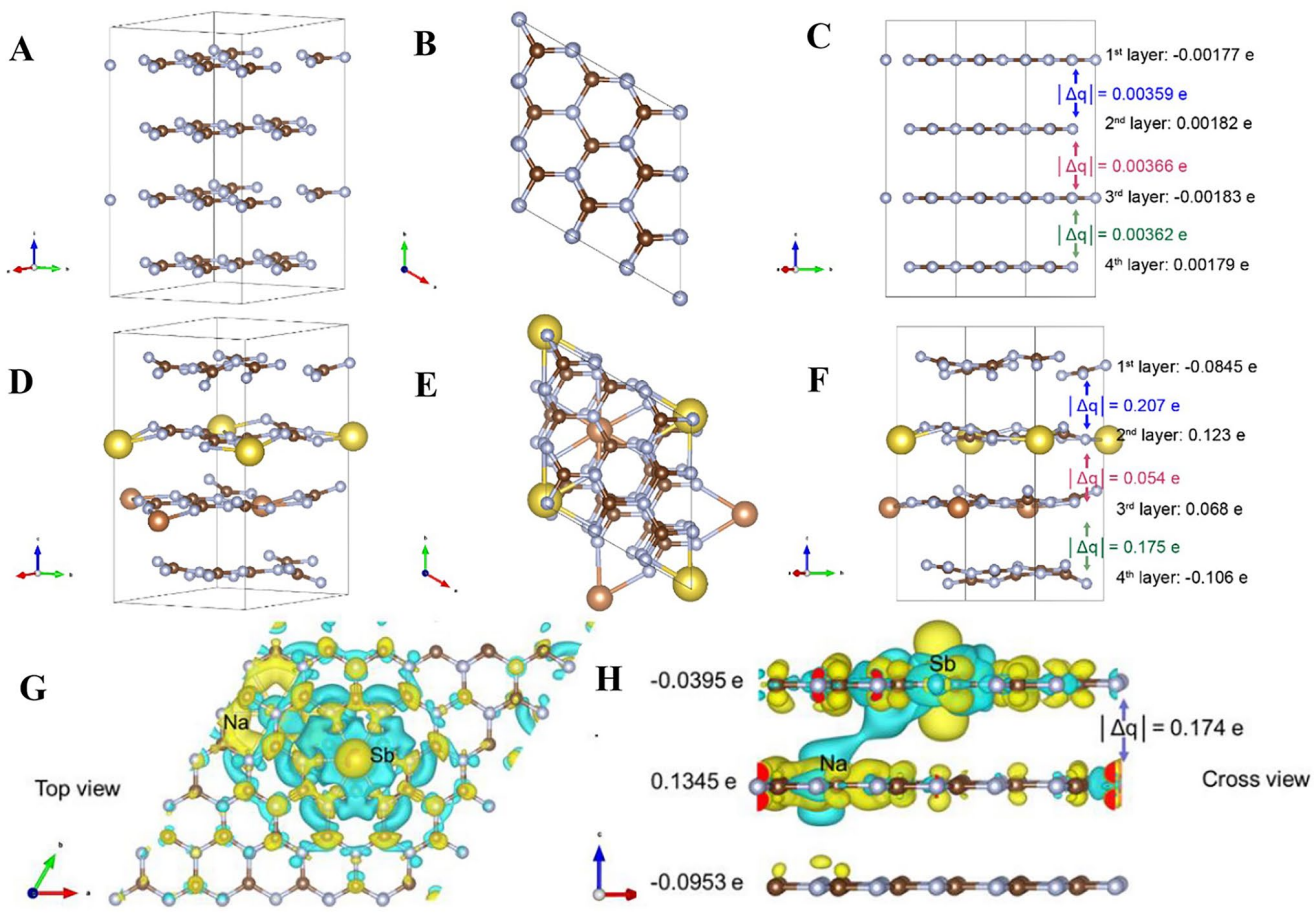
Bader charge distribution analysis from density functional theory (DFT) calculations. **A** Charge distribution of pristine g-C_3_N_4_ (A–C), NaSb/g-C_3_N_4_ (D–F). ǀΔqǀ represents the absolute value of the difference of the electron distribution between the layers; Charge distribution analysis near surface of NaSb-GCN from DFT calculations, **B** enlarged top view and **C** cross view of NaSb-GCN, ǀΔqǀ represents the absolute value of the difference of electron distribution between the first and second layer. Yellow colour represents electron accumulation and blue colour represents electron depletion. Reproduced with permission [107], Copyright 2021, Springer Nature. (Color figure online)

Many photocatalysts, like g-C_3_N_4_, possess a layered structure where the layers are held together by weak van der Waals forces. These weak interactions result in a very weak adiabatic coupling between the layers, which inhibits the transfer of charge carriers between the layers. Teng et al. develop single atom Sb catalysts to improve the inner and interlayer charge transfer of g-C_3_N_4_(GCN) [107]. In the g-C_3_N_4_ structure, the Bader charge difference (ǀΔqǀ) between each adjacent layer is minuscule (roughly 0.004 e^−^ in Fig. 9C), which indicates very weak adiabatic coupling between the layers and results in poor interlayer charge transfer. However, the introduction of Na and Sb atoms into the GCN structure (forming NaSb-GCN) results in a more balanced distribution of electrons across the layers, as the electron density polarization induced by each atom counterbalances each other. This counterbalance decreases the charge difference (ǀΔqǀ to roughly 0.05 e^−^ in Fig. 9F), while simultaneously increasing the distance for adiabatic coupling. The increase in adiabatic coupling distance signifies that charge transfer between the layers is more efficient in NaSb-GCN than in pristine GCN. Regarding the inner layer charge transfer, the deformation charge density near the surface of NaSb-GCN exhibits a clear pathway from Na to Sb, with the first layer showing more electron accumulation than the second layer. The Sb on the surface of GCN, with weak interlayer bridging, leads to a clear region of electron accumulation at the first layer and electron depletion at the second layer (Fig. 9G). The third layer, a pristine CN layer, is barely polarized, which implies that the inner layer charge transfer improves substantially with the incorporation of Na and Sb (Fig. 9H). In conclusion, the DFT calculations show that introducing Na and Sb species into GCN promotes both interlayer and inner layer charge transfers, which can be attributed to the higher photocatalytic activities of Sb-SAPC. The improvement in charge separation and transfer is crucial for photocatalytic reactions as it reduces recombination rates and enhances reaction efficiency.

**Fig. 10:**
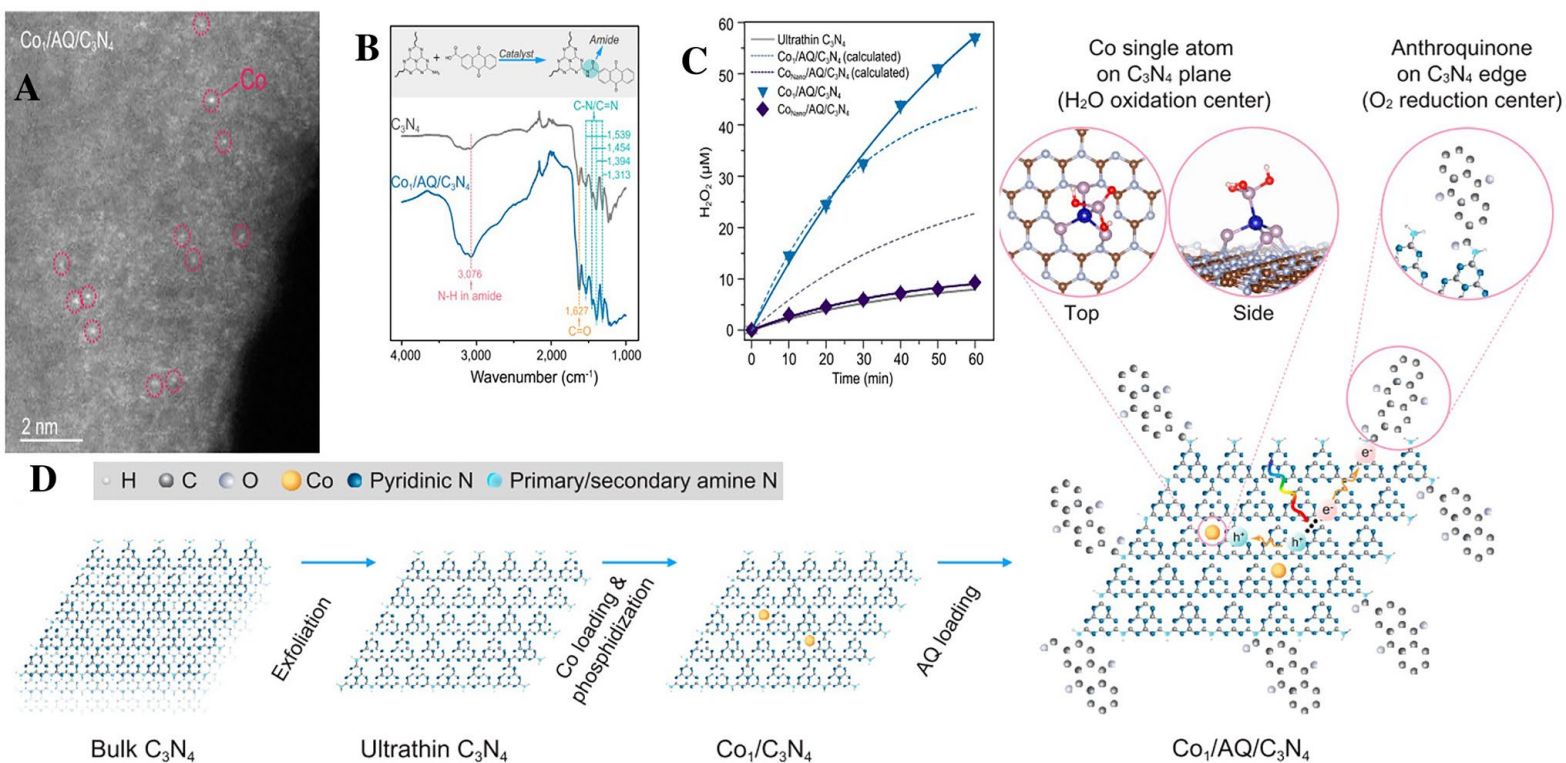
**A** HAADF-STEM image of g-C_3_N_4_ and Co_1_/AQ/g-C_3_N_4_. **B** FT-IR spectra of g-C_3_N_4_ and Co_1_/AQ/g-C_3_N_4_. **C** Time course of H_2_O_2_ production measured under simulated sunlight irradiation (xenon lamp solar simulator, 100 mW cm^−2^, AM 1.5G) with 0.5 g L^−1^ of catalyst under O_2_-saturated condition. **D** Spatial separation of Co single atom (as oxidation center) and AQ (as reduction center) cocatalysts by anchoring them in the center (i.e., pyridinic N) and on the edge (i.e., primary/secondary amine N) of 2D ultrathin g-C_3_N_4_, respectively. Reproduced with permission [26], Copyright 2020, PANS

Single atoms, due to their diminutive size, can be spatially well-isolated within the photocatalyst material. This spatial localization diminishes the likelihood of charge carrier diffusion to recombination sites, thereby bolstering the probability of charge separation. In essence, the presence of single atoms as discrete sites helps to maintain the spatial separation of charge carriers. Each atom acting as an individual catalyst site allows for a uniform distribution of active sites, facilitating the efficient migration of the charge carriers and avoiding recombination. For example, Chu et al. develop the strategy of spatially separating redox centres on 2D carbon nitride with cobalt single atom to improve photocatalytic H_2_O_2_ production [26]. The catalysts were constructed by utilizing–g-C_3_N_4_ as the base substrate. Single atoms of Co were subsequently deposited onto the central region of the carbon nitride structure, while anthraquinone (AQ) was placed along the edges (Fig. 10A–B). This configuration facilitated the segregation of oxidative and reductive cocatalysts, enhancing the efficiency of the chemical reaction. When cobalt single atoms are loaded onto g-C_3_N_4_, it greatly enhances the photocatalyst's ability to perform water oxidation. This is an important step in the process of photocatalytic H_2_O_2_ production. Water oxidation is the reaction where water is broken down into oxygen, hydrogen ions (protons), and electrons. This is evident from an 8.4-fold enhancement in oxygen production over 4 h (Fig. 10C). This enhancement is attributed to strong adsorption of water molecules on the single atom of Co, according to DFT calculations. Further, the Co atom introduces new energy states in the bandgap of g-C_3_N_4_, facilitating efficient charge separation. AQ is loaded onto g-C_3_N_4_ as a second co-catalyst. DFT calculations confirmed the role of AQ in electron withdrawal, with the AQ molecule generating an empty state close to the conduction band (CB) of g-C_3_N_4_. AQ prevents the transfer of a photoexcited hole, allowing only the transfer of a photoexcited electron, leading to enhanced charge separation.

**Fig. 11:**
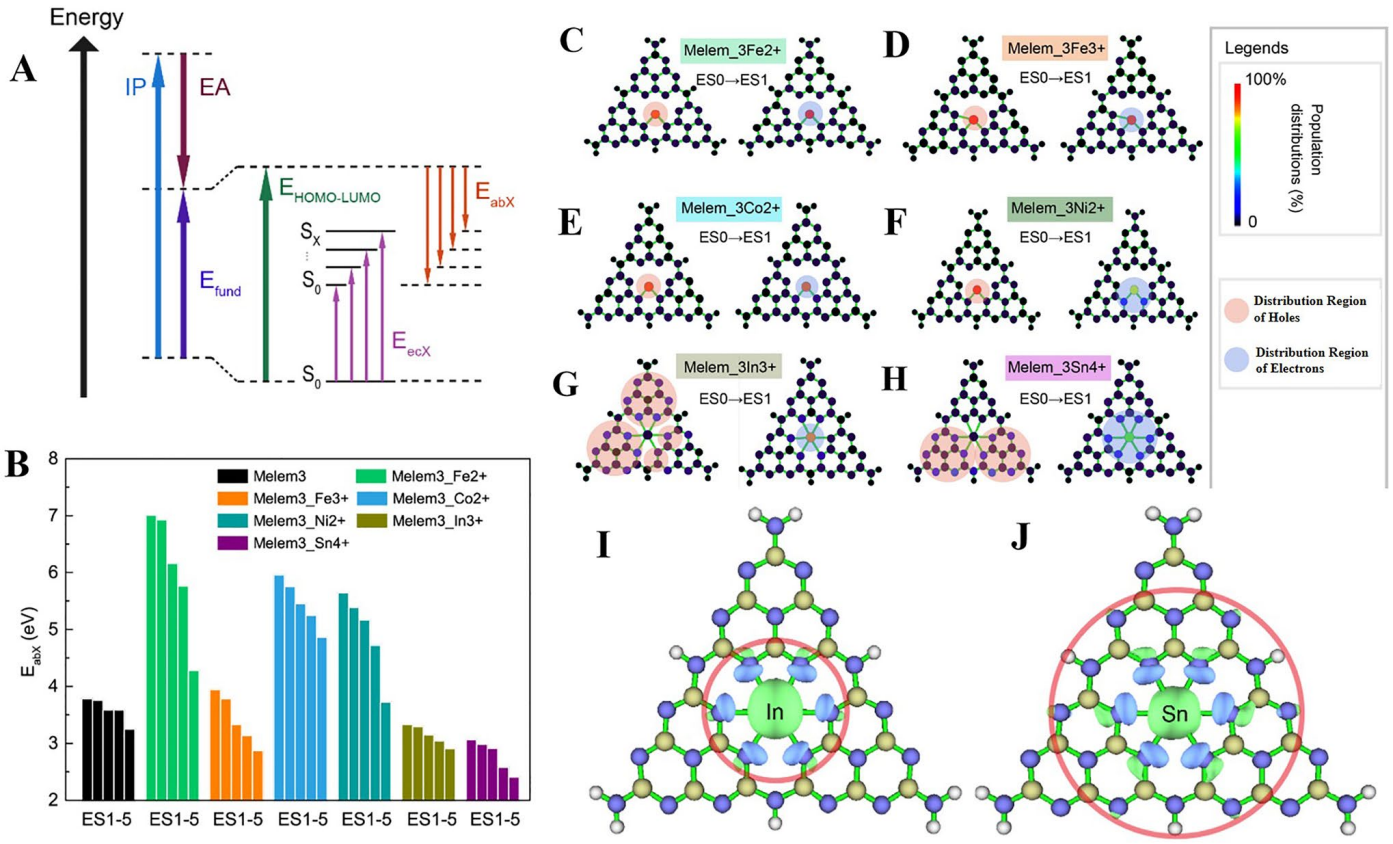
**A** Illustration of gap energies in the molecular case: S0 denotes the electronic ground state and SX is the No. X excited state. **B** The computed approximate binding energies of Melem_3 and Melem_3M for the five lowest-lying excited states; Population of electron and hole distributions (vertical excitation at the excited state 1) and quantitative investigation of the charge separation. The color in the heatmap refers to the sum of MO contribution at each atom for simulated electrons and holes of **C** Melem_3Fe^2+^, **D** Melem_3Fe^3+^, **E** Melem_3Co^2+^, **F** Melem_3Ni^2+^, **G** Melem_3In^3+^, and **H** Melem_3Sn^4+^; Visualization of the dominant contributing molecular orbitals and transition. Visualization of the LUMOs of **I** Melem_3In^3+^ and **J** Melem_3Sn^4+^. Reproduced with permission [117], Copyright 2021, Elsevier

The electronic configuration of single atom catalysts also plays a pivotal role in charge separation during photocatalytic reactions. The electronic configuration determines the energy levels of the valence and conduction bands. A suitable alignment of these energy levels with respect to the redox potentials of the reactants is essential for charge separation. If the electronic configuration allows the metal atom to accept photo-generated electrons easily, it will contribute to efficient charge separation. For example, Teng et al. develop a set of metal single-atom photocatalysts, whereby three non-noble metals (Fe, Co, Ni) and two main-group metals (In, Sn) were individually incorporated as single sites into the polymeric carbon nitride (PCN) framework through pyridinic N atoms [117]. They use the approximate electron–hole pair binding energy (E_abX_) to estimate the tendency of charge recombination. The E_abX_ values for Melem_3In^3+^ and Melem_3Sn^4+^ are found to be smaller than for Melem_3, suggesting that the introduction of In^3+^ and Sn^4+^ into the g-C_3_N_4_ units could suppress charge recombination (Fig. 11A–B). In contrast, the incorporation of Fe^2+^, Fe^3+^, Co^2+^, and Ni^2+^ caused a significant increase in E_abX_, indicating a tendency for thermodynamically favoured recombination. Suppression of charge recombination allows for more efficient catalytic activity because separated charge carriers can participate in redox reactions. Besides, the distribution of photo-generated electrons and holes (electronic transition densities) of Melem_3M are analysed. For Melem_3Fe^2+^, Fe^3+^, Co^2+^, and Ni^2+^, both the electrons and holes are distributed in a very small region just near the metal sites, indicating localized transitions (Fig. 11C–F). For Melem_3In^3+^ and Melem_3Sn^4+^, however, the transitions are dominated by charge transfer excitations, which are mostly associated with the In and Sn atoms (Fig. 11G–H). This suggests a better spatial separation of charges, which is crucial for the photocatalytic activity. Moreover, the contribution of molecular orbitals (MOs) to holes and electrons revealed that the LUMO (Lowest Unoccupied Molecular Orbital) is dominant for electrons in In^3+^ and Sn^4+^, suggesting that the electrons are more likely to accumulate at these atomic sites (Fig. 11I–J). This was confirmed by the iso-surface plots of LUMO, which showed a high concentration of electrons at the In and Sn sites, leading to an ideal electronic configuration for the adsorption of electrophilic oxygen and therefore possibly accelerating the ORR.

In conclusion, the enhancement of photocatalytic reactions depends on the interplay of various factors such as catalyst structure, charge separation and transfer, and the electronic configuration of the catalysts. The use of single-atom catalysts provides a path to optimizing these factors due to their unique properties. The incorporation of single atoms, such as Sb, into a g-C_3_N_4_ structure can improve charge transfer between the layers and within a layer, contributing to improved photocatalytic activity. These elements counterbalance each other's electron density polarization, thus reducing the charge difference and enhancing charge transfer efficiency. Furthermore, single atoms are spatially well-isolated within the photocatalyst material, minimizing the chance of charge carrier diffusion to recombination sites, thereby increasing the probability of charge separation. This attribute facilitates the segregation of oxidative and reductive cocatalysts, enhancing the efficiency of chemical reactions and boosting photocatalytic H_2_O_2_ production. Lastly, the electronic configuration of the single atom catalysts plays a significant role in the charge separation during photocatalytic reactions. The right alignment of energy levels, facilitated by the chosen atomic species, is crucial for efficient charge separation, thus leading to better spatial separation of charges and suppressing charge recombination. This is essential for improving the photocatalytic performance and the overall reaction efficiency. The studies also suggest that the electronic configuration determines the concentration of electrons at atomic sites, which in turn can influence the adsorption of electrophilic oxygen and possibly accelerate the ORR. This comprehensive approach, combining material design, charge dynamics, and electronic configuration, provides a promising avenue for advancing photocatalysis science.

### Improving the Adsorption and Activation of Reactants

For efficient photocatalytic H_2_O_2_ production, it is crucial to lower the energy barriers of reactant adsorption and activation. This section examines how single atoms contribute to achieving this goal, making the entire photocatalytic process more energy-efficient and effective.

**Fig. 12:**
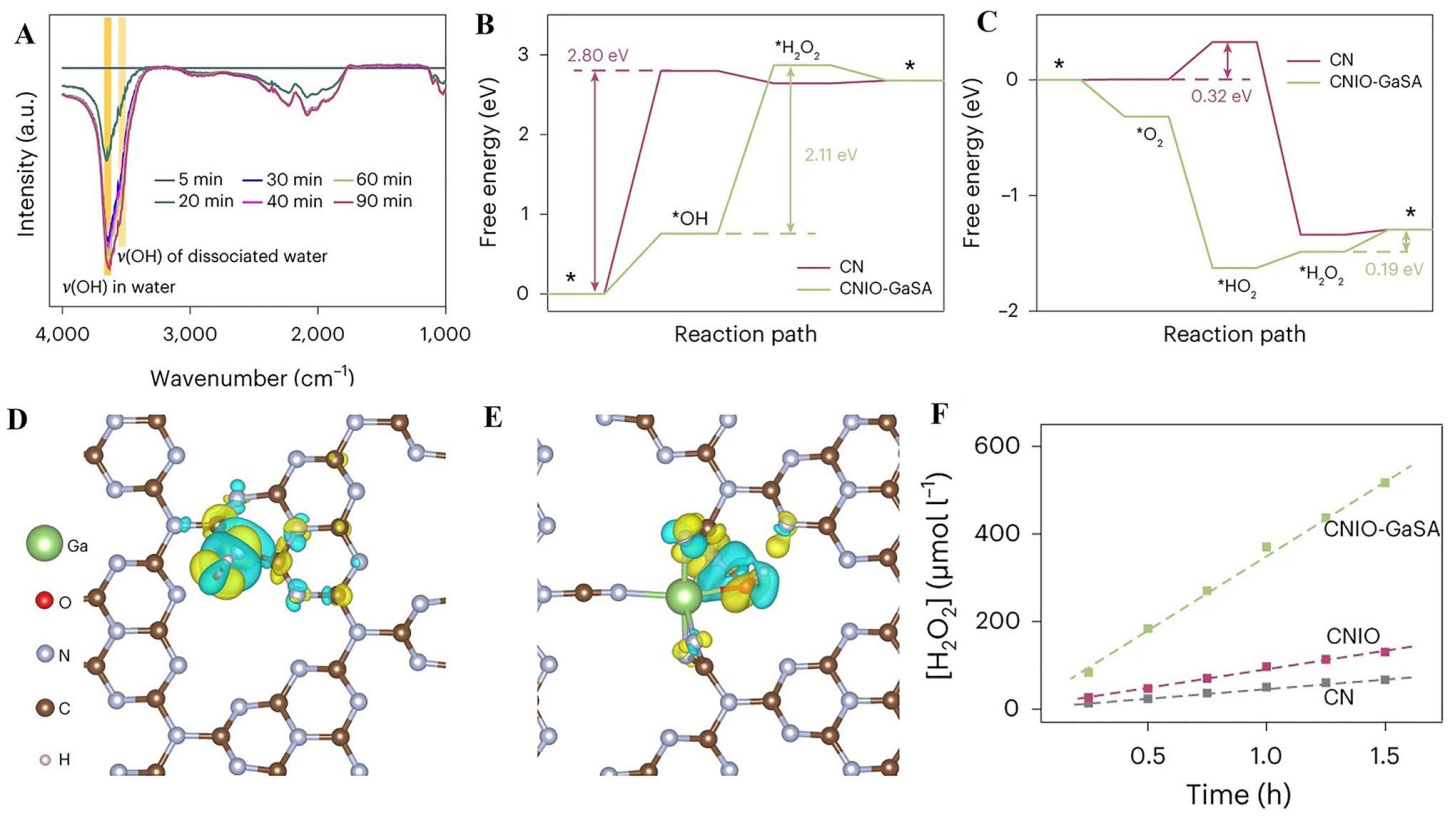
**A** In situ FTIR spectrum analysis of the H_2_O_2_ production over CNIO-GaSA and O dissociation process over CNIOGaSA. **B** Oxidation of water into H_2_O_2_ and **C** Reduction of oxygen into H_2_O_2_ on pristine CN and CNIO-GaSA at electric potential U = 0 V versus SHE at pH 7. **D** Charge density mapping between *OH group and pristine CN photocatalysts. **E** Charge density mapping between *OH group and CNIO-GaSA photocatalysts. **F** Time profiles of H_2_O_2_ photoproduction by various CN photocatalysts. Experimental conditions: photocatalyst (1 g L^–1^, 2 mL) under visible-light irradiation (λ ≥ 420 nm, 100 mW cm^–2^), 1 atm O_2_ and T = 303 K. Reproduced with permission [41], Copyright 2023, Springer Nature

The orbital hybridization of single atom catalysts can significantly influence the adsorption and activation of reactants, affecting the overall performance and efficiency of the catalyst. The specific effects depend on the nature of the hybridization and the specific reactants involved. The ability of a single atom catalyst to adsorb a reactant—that is, to have the reactant attach to the catalyst's surface—is heavily influenced by the catalyst's electronic structure, which is determined by its orbital hybridization. The type of hybridization (*sp*, *sp*^2^, or *sp*^3^) impacts the distribution of electrons around the atom, and therefore, it affects how the catalyst can form weak bonds (adsorb) with reactants. Activation of reactants generally involves the weakening, breaking, and formation of chemical bonds. Orbital hybridization influences the energy levels and spatial arrangement of the catalyst's electrons, which can affect how effectively it can overlap with the orbitals of the reactant to form new bonds. Effective orbital overlap can lower the energy barrier for a reaction, thereby improving the efficiency of reactant activation. For example, Tan et al. develop the strategy of a Ga–N_5_ atomic site on macroporous inverse-opal-type carbon nitride (CNIO-GaSA) for photocatalytic hydrogen peroxide production [41]. They discover that the hybridized Ga 4*p* and N 2*p* states promote the dissociation of water molecules on the surface of CNIO-GaSA, leading to the formation of the *OH intermediate (Fig. 12A). This *OH intermediate is crucial for the two-electron WOR and ORR, key steps in H_2_O_2_ production. The formation of *OH or *OOH on CNIO-GaSA has a lower energy barrier than on pristine CN (Fig. 12B–C). This is because, in the CNIO-GaSA system, *OH can bind with both the Ga single atom and the neighbouring nitrogen atom via Ga–O and N–O bonds, facilitated by the hybridized Ga 4*p* and N 2*p* states (Fig. 12D–E). This bond formation effectively reduces the energy barrier for the WOR, enhancing the overall production of H_2_O_2_ (Fig. 12F).

**Fig. 13:**
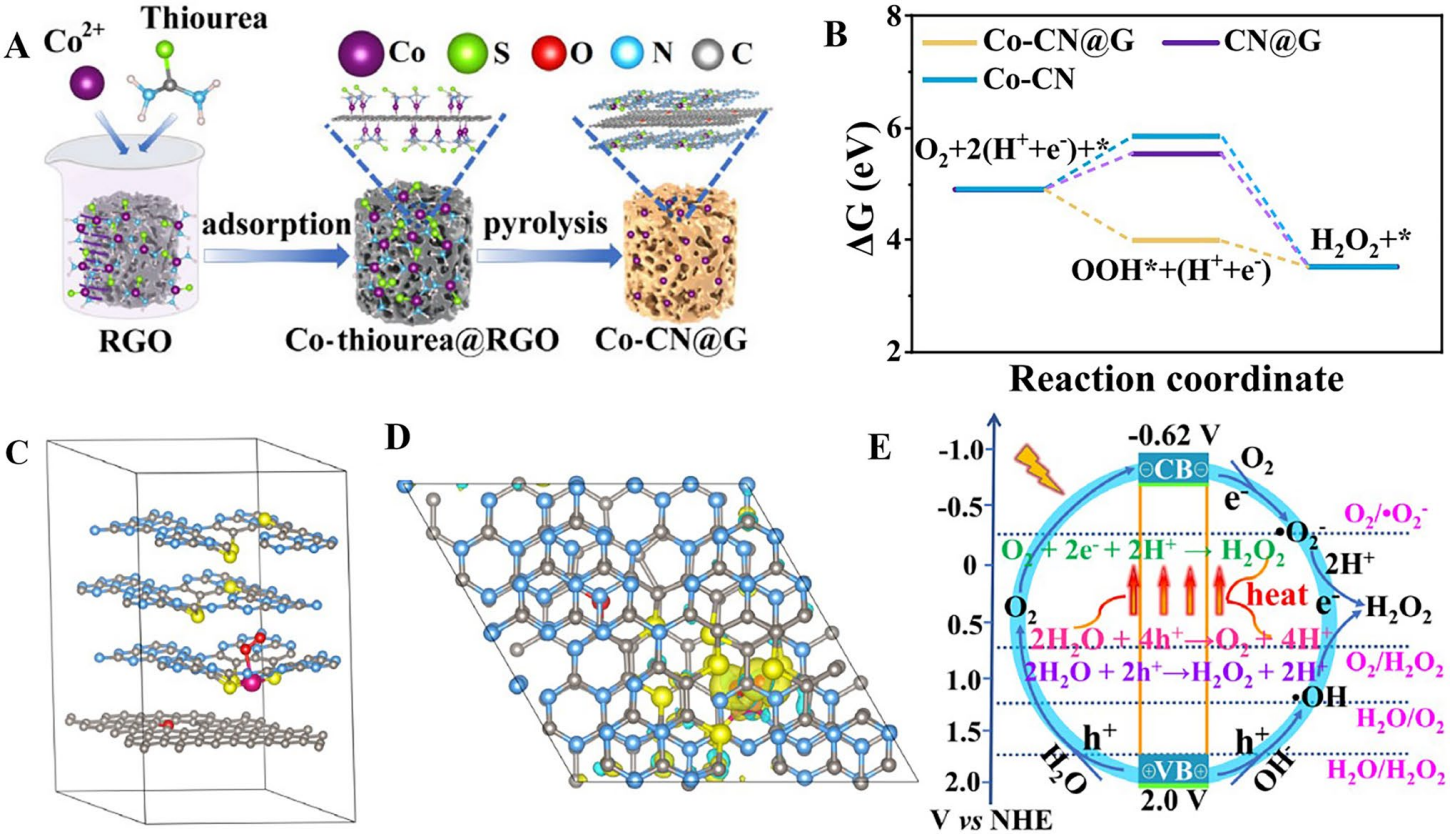
**A** Schematic illustration of the synthesis of Co–CN@G. **B** Free energy diagram for H_2_O_2_ formation through the O_2_ reduction pathway. **C** Configuration of O_2_ adsorption on Co–CN@G from a cross-sectional perspective. **D** Top view of the charge difference density during O_2_ adsorption on Co–CN@G. The yellow and cyan iso-surfaces indicate electron accumulation and depletion, respectively. Nitrogen, carbon, sulphur, cobalt, and oxygen are represented by the colours blue, grey, yellow, rose, and red, respectively. **E** Mechanism of photothermal-photocatalytic H_2_O_2_ production. Reproduced with permission [40], Copyright 2023, Springer Nature. (Color figure online)

The coordination environment around a single atom, including its immediate neighbouring atoms and the corresponding electronic structure (referred to as the “coordination band”), can significantly influence the performance of SACs in catalytic reactions. The coordination environment determines the electronic structure of the single atom and therefore its ability to adsorb reactants. Different coordination environments may favour or hinder the adsorption of specific reactants. By fine-tuning the coordination environment, it's possible to optimize the binding strength between the catalyst and reactants, which is critical for catalytic performance. For example, Zhang et al. synthesize sulfur doped graphitic carbon nitride/reduced graphene oxide heterostructure (Co–CN@G) confined single Co atoms with N/S coordination for high H_2_O_2_ production (Fig. 13A) [40]. It is found the energy associated with the adsorption of O_2_ on Co–CN@G is negative (− 0.28 eV), which means that this process releases energy and is exothermic (Fig. 13C). Moreover, this energy is significantly lower compared to when Co is not present as single atoms, which makes the O_2_ adsorption more efficient on Co–CN@G. On Co–CN@G, cobalt atoms form bonds with oxygen (Co–O) that are shorter compared to similar structures without single-atom Co. This leads to a stretching of the O=O bond in the O_2_ molecule. These bond configurations allow for stronger interactions between the O_2_ and the structure, which is beneficial for the activation of O_2_. There is notable accumulation of electrons on the oxygen atoms bonded to cobalt, while the cobalt atoms have electron depletion (Fig. 13D). This indicates that electrons are being transferred from the Co atoms to the oxygen atoms (known as back-donation). These extra electrons on O_2_, particularly from the photogenerated electrons on Co–CN@G, can activate the O_2_ by entering its anti-bonding orbitals. This makes the O_2_ molecule more reactive, which is crucial for its conversion to H_2_O_2_. With O_2_ activated, the system efficiently drives the reduction of O_2_ in a step-wise manner, with O_2_ being reduced and gaining electrons (Fig. 13E). This process is energetically favourable (exothermic) in the presence of Co–CN@G and leads to the formation of H_2_O_2_. The Co–CN@G is also more efficient in promoting water oxidation as part of the H_2_O_2_ production process. This is evidenced by a lower energy requirement for the generation of a critical intermediate state (OH*), as compared to other catalysts (Fig. 13B). In summary, the single Co atoms integrated into the CN@G heterostructure play a crucial role in facilitating the adsorption and activation of O_2_. This, in turn, lowers the energy barriers involved in O_2_'s reduction to H_2_O_2_ and water oxidation, leading to an enhanced efficiency in photocatalytic H_2_O_2_ production, as supported by both the theoretical calculations and experimental results.

In summary, single atom catalysts, through enhanced electron delocalization and unique bond configuration, can significantly lower the energy barriers for reactant adsorption and activation. By doing so, they contribute to optimizing photocatalytic efficiency, making single atom catalysts highly promising for improving photocatalytic H_2_O_2_ production. Future research should continue to explore these promising properties, seeking new ways to design and utilize single atom catalysts for even greater energy efficiency and reactivity in photocatalysis.

## Challenges and Future Directions

### Challenges of Single Atom Catalysts in Hydrogen Peroxide Photosynthesis

#### Hydrogen Peroxide Decomposition

In single atom photocatalysis aimed at H_2_O_2_ production, a noteworthy challenge lies in circumventing the inadvertent decomposition of the synthesized H_2_O_2_. This is especially relevant considering the intrinsic reactivity of single atom catalysts [27, 118–120]. While the active sites of these catalysts are advantageous for H_2_O_2_ synthesis, they may concurrently facilitate its degradation, consequently diminishing the overall yield [121, 122]. Hydrogen peroxide decomposition is governed by two principal mechanisms: homolytic cleavage and photocatalytic decomposition. Homolytic cleavage involves the splitting of the H_2_O_2_ molecule into two hydroxyl radicals (•OH) as described by the reaction H_2_O_2_ → 2⋅OH. Being highly reactive, these hydroxyl radicals can engage in various secondary reactions, leading to further decomposition of H_2_O_2_. In the context of photocatalytic decomposition, catalysts such as TiO_2_ play a pivotal role. Upon absorption of photons, the catalyst promotes the excitation of electrons to a higher energy state, enabling them to react with H_2_O_2_. This leads to the disintegration of H_2_O_2_ into water and oxygen, represented by the Eq. 2H_2_O_2_ → 2H_2_O + O_2_. This photocatalytic pathway poses a challenge in the synthesis of H_2_O_2_, as it detracts from the overall yield of the target product, introducing inefficiencies into the production process.

The goal is to engineer catalysts that can increase H_2_O_2_ production while minimizing its decomposition. This necessitates a balance in reactivity: active enough to drive the desired reaction but not so active as to cause product decomposition. This balance demands an intricate understanding of the reaction mechanisms, as well as precise control over the electronic structure and the coordination environment of the single atoms. Controlling reaction conditions to favour H_2_O_2_ production is also essential, involving the adjustment of variables such as light intensity, reaction temperature, pH, and concentrations of reactants. It might also be advantageous to explore strategies to stabilize produced H_2_O_2_, like the use of additives or developing systems that rapidly remove H_2_O_2_ from the reaction site. Although the issue of H_2_O_2_ decomposition is challenging, it presents an opportunity for advancements and innovation in single atom photocatalysis.

#### Stability of Single Atom Catalysts

In the realm of single atom photocatalysis for H_2_O_2_ production, stability stands as a primary concern. Structural changes such as aggregation or sintering can cause single atom catalysts to lose their active sites [123–125]. Under conditions of high temperatures, harsh chemicals, or prolonged use, these changes might be triggered, compromising the catalyst's efficiency. The bonding strength between the atom and its host material is critical for maintaining stability. Furthermore, catalyst fouling by reaction intermediates or impurities can deactivate the catalyst and shorten its lifespan [126–128].

To overcome these challenges, new approaches in catalyst design are needed. Stronger interactions between single atoms and the host material could prevent displacement or aggregation. The use of protective agents might guard against poisoning or fouling, while self-healing or regenerative abilities could restore the catalyst's structure and activity after deactivation. Optimizing the stability of single atom catalysts involves a mix of materials design, perfecting reaction conditions, and employing innovative strategies to ensure durability in H_2_O_2_ production. Future research should focus on discovering new materials and techniques to enhance catalyst stability.

#### Unclear Mechanism of Hydrogen Peroxide Photosynthesis over Single Atom Catalysts

Understanding the mechanics behind the effectiveness of SACs in photocatalytic H_2_O_2_ production remains a challenge [129–131]. Elements like light absorption, charge separation, and surface reactions introduce complexity into the process. While SACs are known to modify band structure and boost light absorption, more research is needed to grasp the interplay between photogenerated charge carriers and single atoms, and to fully understand charge dynamics [132, 133]. Clarification is also needed on how SACs impact charge migration and surface reactions, and how their surrounding environment affects catalytic properties.

Unveiling these uncertainties necessitates a blend of practical and computational methods. *In-situ* and operando techniques, such as X-ray absorption spectroscopy, electron paramagnetic resonance, and operando Raman spectroscopy, can offer real-time data on SACs behaviour, while theoretical calculations can model electronic structures and predict reaction pathways. Isotope labelling experiments can help track reactants, intermediates, and products, thus elucidating the H_2_O_2_ synthesis mechanism. The expansion of characterization techniques, theoretical models, and continuous research will be key in unravelling the complex reactions involved in SACs-catalysed processes. This knowledge will help maximize the potential of SACs in photocatalytic H_2_O_2_ production and other energy-related applications.

#### Low Selectivity

SACs have great reactivity but face challenges in selectively producing H_2_O_2_ due to competition with other reactions, such as water splitting [1, 134–136]. This low selectivity can be attributed to the catalyst's environment which, if not properly designed, may either fail to promote H_2_O_2_ production or encourage its degradation [137, 138].

To address this, it's essential to tailor SACs to selectively adsorb and activate oxygen and protons for H_2_O_2_ production. This involves choosing appropriate metal atoms, host materials, and ligands. Also, tweaking reaction conditions like temperature and pH can impact selectivity. Employing kinetic control makes the desired pathway more likely over competing reactions. In addition, adding modifiers to the catalyst can steer the reaction towards H_2_O_2_ production. For instance, using alkaline earth metal cations as promoters can be beneficial. Utilizing advanced techniques to analyse the structure and attributes of SACs and employing computational methods like DFT for studying reaction mechanisms, can offer insights into factors that affect selectivity. This information can guide the creation of more selective catalysts. By tackling the low selectivity issue, H_2_O_2_ production rates can be enhanced while reducing unwanted by-products, making SACs more efficient and feasible for industrial use.

### Future Research Directions of Single Atom Catalysts in Hydrogen Peroxide Photosynthesis

#### Direct Utilization of Hydrogen Peroxide

One strategy to improve single atom photocatalysts' efficiency is to directly use the produced hydrogen peroxide [137, 139, 140]. As a powerful oxidizing agent, H_2_O_2_ is widely used in environmental cleaning, disinfection, and chemical synthesis [141]. Direct use of H_2_O_2_ can increase the practical and economic feasibility of these catalysts [142].

Integration with H_2_O_2_-demanding applications allows immediate use of the product. For instance, these catalysts can be used in wastewater treatment where produced H_2_O_2_ directly degrades pollutants. Another strategy is in-situ usage in tandem reactions, using the produced H_2_O_2_ as a reactant in a subsequent process. Such a "one-pot" reaction can streamline the entire processes, conserving energies and resources. H_2_O_2_ can also be used directly as an oxidant in fuel cells, such as direct borohydride fuel cells. This requires designing catalysts compatible with the operating conditions of these fuel cells. Creating catalysts that work efficiently under conditions allowing for H_2_O_2_'s direct use is crucial. This involves understanding catalysts' stability and reactivity, designing reactors for tandem reactions, and combining knowledge from materials science, chemistry, environmental science, and chemical engineering. Overall, directly using H_2_O_2_ produced by single atom photocatalysts is a promising strategy, enhancing their practicality and economy. Further research in this area could significantly advance the field of single atom photocatalysis.

#### High-throughput Synthesis and Screening

High-throughput synthesis and screening can speed up the development of single atom catalysts for photocatalytic hydrogen peroxide production [143, 144]. This approach allows for fast production and evaluation of many catalysts, saving time and resources. High-throughput synthesis methods, using techniques like atomic layer deposition, can create a wide variety of catalysts by changing factors like metal atom type and host material [145].

Alongside synthesis, high-throughput screening evaluates the catalysts' performance in H_2_O_2_ production. Techniques such as microarray testing and automated data analysis assess multiple catalysts under identical conditions, providing comparative performance data. Additionally, machine learning and data-driven approaches can enhance catalyst development. Machine learning predicts performance based on catalyst features, while data-driven strategies can reveal correlations between catalyst properties and performance, accelerating the discovery of superior catalysts. However, successful implementation needs collaboration across different research areas, development of robust protocols, and advanced tools for handling and analysing the large amounts of data generated from high-throughput experiments.

#### Overall Hydrogen Peroxide Photosynthesis

Improving the efficiency and selectivity of single atom photocatalysts may involve the creation of dual active sites designed for ORR and WOR [107]. This design could make use of unique electronic structures and reactivity of different single atom sites, making photocatalysis more effective [146].

The idea is to separate the two half reactions of H_2_O_2_ synthesis—ORR and WOR—at specialized active sites [147, 148]. This approach could potentially mitigate undesired reactions and improve overall efficiency. The ORR site should bind oxygen molecules optimally for their reduction to H_2_O_2_. The site needs a suitable environment and electronic structure for this process. The WOR site should be optimized for water oxidation to oxygen. It needs a high binding energy for water molecules and the power to break O–H bonds. Synergistic effects between these sites could enhance H_2_O_2_ production efficiency. Separating ORR and WOR could also reduce H_2_O_2_ decomposition and improve reaction selectivity. However, challenges exist, such as controlling the electronic structure, environment at each site, and their spatial arrangement for synergy while minimizing unwanted interactions. Despite the challenges, this dual-site strategy could greatly improve the photocatalytic production of H_2_O_2_ with single atom catalysts.

#### Investigating the Mechanisms of Single Atom Catalysts in Hydrogen Peroxide Photosynthesis Using DFT

DFT, a computational method predicting atomic and molecular behaviour, can help investigate mechanisms of H_2_O_2_ photosynthesis over single atom catalysts. It can offer pivotal insights into fundamental catalytic processes.

DFT can analyse reactant adsorption and activation on single atom catalysts, identify probable reaction pathways, and related energy barriers [40, 149]. Calculating interaction energies and transition states reveals specific roles of single atoms in catalysis, providing theoretical basis for improved catalyst design. Moreover, DFT can help understand charge carrier dynamics in single atom catalysts, predicting behaviour of photogenerated electrons and holes, contributing to catalyst performance optimization [107]. It can also aid in interpreting experimental data, predicting impact of variations in metal atom type, host material, or coordination environment. However, DFT has its challenges. These include accurately capturing electronic correlation effects, especially in transition metal atoms, and modelling complex catalytic environments. Overcoming these needs development of advanced DFT methods and high-performance computing. In the future, the integration of DFT with other computational techniques, such as molecular dynamics or machine learning, could yield more comprehensive and accurate simulations of catalytic processes. This integration can refine the design principles for single atom photocatalysts, thereby facilitating their application in photocatalytic H_2_O_2_ production.

## Conclusion and Outlook

To sum up, SACs represent a promising frontier in the sustainable production of hydrogen peroxide. However, several challenges exist, such as the unintentional decomposition of hydrogen peroxide, the stability of SACs, understanding the exact mechanisms of hydrogen peroxide photosynthesis over these catalysts, and the issue of low selectivity. Tackling these hurdles demands an intricate grasp of reaction mechanisms, meticulous control over the electronic structure of SACs, and attaining a delicate equilibrium in reactivity. Furthermore, innovative strategies such as the use of additives or systems that rapidly remove hydrogen peroxide from the reaction site can be explored to minimize product decomposition. Future research directions are abundantly clear and multi-faceted, including the direct utilization of produced hydrogen peroxide, high-throughput synthesis and screening of SACs, overall improvement in hydrogen peroxide photosynthesis, and the use of computational methods like DFT for investigating the mechanisms of SACs. This research is pertinent not only to material science and chemistry but also carries profound implications for environmental science and chemical engineering.

Despite the challenges faced, the potential of SACs is immense. With continued research and a concerted multidisciplinary approach, they could prove instrumental in realizing the dream of sustainable hydrogen peroxide production. This would mark a significant stride in the field of green chemistry and environmentally friendly applications, thereby reinforcing the pivotal role of SACs in our sustainable future.
